# Synthesis of Curcumin Derivatives via Knoevenagel Reaction Within a Continuously Driven Microfluidic Reactor Using Polymeric Networks Containing Piperidine as a Catalyst

**DOI:** 10.3390/gels11040278

**Published:** 2025-04-08

**Authors:** Naresh Killi, Katja Rumpke, Dirk Kuckling

**Affiliations:** Department of Chemistry, Faculty of Science, Paderborn University, Warburger Str. 100, 33098 Paderborn, Germany; naresh.killi@uni-paderborn.de (N.K.); krumpke@mail.uni-paderborn.de (K.R.)

**Keywords:** flow chemistry, heterogeneous catalysis, sustainable synthesis, organo-catalysis, polymeric gel dots

## Abstract

The use of organo-catalysis in continuous-flow reactor systems is gaining attention in medicinal chemistry due to its cost-effectiveness and reduced chemical waste. In this study, bioactive curcumin (CUM) derivatives were synthesized in a continuously operated microfluidic reactor (MFR), using piperidine-based polymeric networks as catalysts. Piperidine methacrylate and piperidine acrylate were synthesized and subsequently copolymerized with complementary monomers (MMA or DMAA) and crosslinkers (EGDMA or MBAM) via photopolymerization, yielding different polymeric networks. Initially, batch reactions were optimized for the organo-catalytic Knoevenagel condensation between CUM and 4-nitrobenzaldehyde, under various conditions, in the presence of polymer networks. Conversion was assessed using offline ^1^H NMR spectroscopy, revealing an increase in conversion with enhanced swelling properties of the polymer networks, which facilitated greater accessibility of catalytic sites. In continuous-flow MFR experiments, optimized polymer gel dots exhibited superior catalytic performance, achieving a conversion of up to 72%, compared to other compositions. This improvement was attributed to the enhanced swelling in the reaction mixture (DMSO/methanol, 7:3 *v*/*v*) at 40 °C over 72 h. Furthermore, the MFR system enabled the efficient synthesis of a series of CUM derivatives, demonstrating significantly higher conversion rates than traditional batch reactions. Notably, while batch reactions required 90% catalyst loading in the gel, the MFR system achieved a comparable or superior performance with only 50% catalyst, resulting in a higher turnover number. These findings underscore the advantages of continuous-flow organo-catalysis in enhancing catalytic efficiency and sustainability in organic synthesis.

## 1. Introduction

Natural compounds play a pivotal role in pharmaceutical applications due to their diverse medicinal properties [1,2]. Many of these compounds serve as direct therapeutic agents, including curcumin (CUM), a bioactive polyphenol extracted from the rhizomes of *Curcuma longa*. Traditionally used in Ayurvedic medicine and as a food additive in Southeast Asia, CUM exhibits a broad spectrum of pharmacological activities, including antiviral, antibacterial, antifungal, anticancer, and anti-inflammatory effects [3,4,5]. Despite its therapeutic potential, CUM’s clinical applications are significantly hindered by its poor aqueous solubility, pH instability, limited absorption, rapid degradation, and low bioavailability [6]. To enhance its pharmaceutical efficacy, various strategies have been explored, with chemical modifications proving particularly effective in improving bioavailability and medicinal activity. For instance, water-soluble CUM analogues conjugated with amino acid salts have demonstrated enhanced anticancer potential [7,8]. Similarly, Sahu et al. reported benzylidene CUM derivatives with increased antibacterial and antifungal activity [9]. A widely adopted approach for CUM modification involves the Knoevenagel condensation reaction, catalyzed by piperidine (PD) as an organo-catalyst [10]. This reaction facilitates the formation of novel bioactive agents through C–C bond formation between CUMs active methylene group and various aldehydes. By leveraging these chemical transformations, CUM analogues with improved pharmacokinetic profiles and therapeutic efficacy can be developed, expanding their potential in medicinal chemistry and drug discovery [11].

PD, a widely used organic base, serves as an efficient catalyst in various organo-catalytic transformations, including the Knoevenagel condensation, Mannich reaction, and Michael addition, due to its high catalytic activity [12,13,14]. Traditionally, PD-catalyzed reactions are conducted under homogeneous conditions, necessitating catalyst removal via acid neutralization, which leads to the formation of ammonium salts as by-products. These salts require additional purification steps, complicating the overall process and posing challenges for acid-sensitive functional groups. To circumvent these limitations, heterogeneous catalysis provides an effective alternative, wherein the catalyst and reactants remain in separate phases, allowing for facile catalyst recovery via simple filtration [15,16]. One promising approach involves immobilizing organo-catalysts within insoluble three-dimensional polymer networks, thereby integrating the advantages of both homogeneous and heterogeneous catalysis. For instance, Sobhani et al. reported the immobilization of PD onto silica-coated iron oxide nanoparticles for organo-catalysis [17]. While these catalysts can be recovered via centrifugation, filtration, or magnetic separation, such processes are still often time-consuming and energy-intensive, and they may hinder catalytic-site accessibility [18]. Additionally, residual metal impurities can introduce toxicity concerns, particularly in pharmaceutical applications [19,20,21]. Recent advancements in organo-catalysis emphasize metal-free polymer-immobilized catalysts that exhibit high catalytic activity and improved reusability [22]. Polymer networks, in particular, offer significant advantages due to their high swelling capacity in reaction solvents that enhances catalytic-site accessibility. For example, gel-bounded piperidine-based catalysts are used for synthesis of complex molecules at mild reaction conditions and contribute to green chemistry by reducing toxic solvents and metals [23]. In addition, these catalysts are homogeneously destituted in the reaction mixture, are stable, and can be separated easily after the reaction. Hence, these catalysts are more attractive to industry due to their low cost, higher safety, and high catalytic activity. Given these attributes, PD was selected for immobilization within polymeric networks, with the aim of synthesizing catalytically active monomers and polymerizing them into functional polymer networks for the Knoevenagel reaction, facilitating the synthesis of CUM derivatives [24].

**Figure 1:**
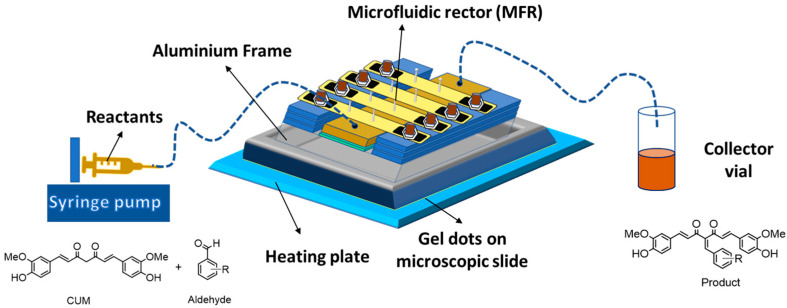
Graphical representation of the MFR system for synthesis of CUM derivatives. Heat transfer was realized by inserting the assembly in an aluminum holder on a hot plate.

Furthermore, microfluidic reactor (MFR) technology has gained prominence in modern chemistry, with applications in protein separation, drug discovery, and organic synthesis [25,26,27]. Microfluidic reactions offer superior sensitivity, efficient heat transfer, and high conversion rates compared to conventional batch reactions, aligning with sustainable and cost-effective green chemistry principles [28,29]. Additionally, continuous-flow processing allows for precise reaction control and scalability. The MFR system typically consists of three main components: a syringe pump, a microfluidic reactor, and a collection vial, interconnected by microchannels to regulate flow (Figure 1). Previous studies have demonstrated that polymer network-based heterogeneous catalysts in continuous-flow reactors significantly outperform batch reactions in terms of conversion efficiency [30,31]. Notably, amine-functionalized polymer networks have been successfully employed as catalysts in the Knoevenagel reaction involving aldehydes and malononitrile [32,33]. Building upon these advancements, the present study aims to synthesize biologically active CUM derivatives within a continuous MFR system. This work involves the design and synthesis of catalytically active monomers (CAM), followed by the fabrication of polymer networks via photopolymerization. These polymer networks are then utilized in continuous-flow reactions within a MFR system, with a focus on evaluating the catalytic activity of different polymer compositions in the Knoevenagel reaction between CUM and various aldehydes. Additionally, the impact of polymer network swelling behavior and reaction temperature on conversion efficiency is systematically investigated.

## 2. Results and Discussion

**Scheme 1:**
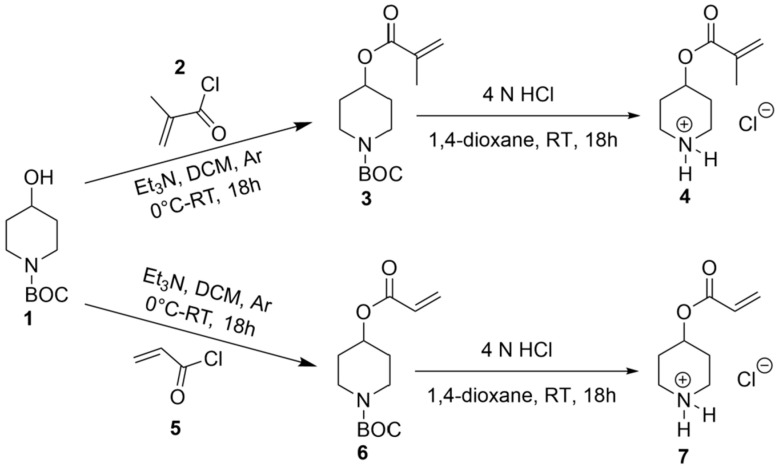
Synthesis of CAMs, 4-(methacryloyloxy) piperidin-1-ium chloride (**4**) and 4-(acryloyloxy) piperidin-1-ium chloride (**7**).

The synthesis of CUM derivatives was carried out via the Knoevenagel reaction, employing PD-based polymeric networks as organo-catalysts. To achieve this, PD-functionalized (meth-)acrylate monomers were synthesized through a nucleophilic substitution reaction between *tert*-butyl-4-hydroxypiperidine-1-carboxylate and methacryloyl or acryloyl chloride in the presence of triethylamine as a base. This reaction yielded *tert*-butyl-4-piperidinyl methacrylate (**3**) and *tert*-butyl-4-piperidinyl acrylate (**6**). Subsequently, deprotection of the amine functionality was performed at room temperature using 4 N HCl in 1,4-dioxane, yielding 4-(methacryloyloxy) piperidin-1-ium chloride (**4**) and 4-(acryloyloxy) piperidin-1-ium chloride (**7**), as illustrated in Scheme 1. The resulting monomers were purified by high-vacuum drying, with an overall reaction yield of 60–65%. The chemical structures of the synthesized monomers were confirmed via NMR spectroscopy, as evidenced by the presence of characteristic (meth-)acrylate and piperidyl proton signals (Supplementary Figures S1 and S2).

**Figure 2:**
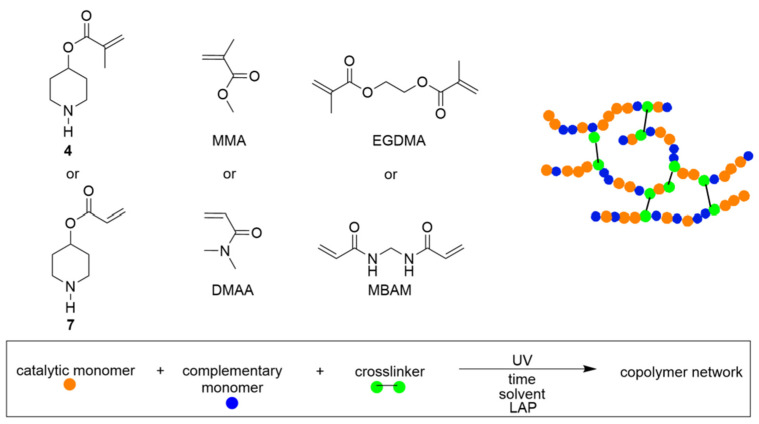
Schematic representation for fabrication of polymer networks using photopolymerization.

The fabrication of PD-based polymeric networks was carried out using the synthesized CAMs (**4**) and (**7**) in combination with a complementary monomer and a crosslinker through photopolymerization. A schematic representation of the polymer network formation process is shown in Figure 2. Initially, various monomer compositions (A–G) were prepared by dissolving CAMs (**4** or **7**) with the complementary monomers—methyl methacrylate (MMA) or *N*,*N*-dimethylacrylamide (DMAA); and crosslinkers—ethylene glycol dimethacrylate (EGDMA) or *N*,*N*-methylenebis(acrylamide) (MBAM)—in water to achieve a total monomer concentration of 19 or 25 mmol mL^−1^. These monomer solutions were poured into a mold and exposed to UV irradiation through a mask under optimized photopolymerization conditions (Table 1 and Table S1 for bulk gels). In all compositions (A–G), structurally compatible monomers were used to ensure the formation of homogeneous copolymer networks. However, compositions C and D failed to form gels due to phase separation of MMA and CAMs at higher ratios.

Following the optimization of photopolymerization conditions, the same monomer compositions were employed to fabricate polymer gel dots using a photomask containing hexagonal arrays of 662 dots. The polymer networks were formed on the surface of modified microscope glass slides, which were functionalized following a previously reported method [33]. Acrylate groups on the glass surface enabled the attachment of polymer gel dots. Further optimization of gel formation was conducted by varying the UV irradiation time to ensure high-quality polymer gel dots. The final composition of the polymer networks and their polymerization parameters are detailed in Table 1. Upon synthesis, the polymeric gel dots were washed with methanol and subsequently neutralized using a triethylamine/methanol solution (1:9, *v*/*v*) to eliminate hydrochloride salts from the polymer matrix. The neutralized gel dots were then dried and directly utilized as catalysts in the MFR for performing the Knoevenagel reaction between different aldehydes and CUM.

The swelling behavior of polymeric networks plays a critical role in catalysis within the MFR. Swollen polymer gels create a porous, mesh-like structure in the reaction solvent, enhancing the accessibility of catalytic sites and consequently improving reaction conversion. To evaluate these properties, the swelling capacity of cylindrical polymer gels was studied in a reaction solvent mixture of DMSO and methanol (7:3, *v*/*v*) at both room temperature and 40 °C for 2 h and 24 h. The selection of the solvent system was based on the solubility of the starting material, CUM, in various solvents (Supplementary Table S2). Among the tested systems, DMSO/methanol (7:3, *v*/*v*) provided a clear solution, ensuring optimal reaction conditions. Additionally, the polymer gels showed superior stability and swelling properties in methanol compared to water and ethanol (Supplementary Table S3).

Cylindrical polymer gels were fabricated from monomer compositions A, B, E, F, and G using a Pasteur pipette as a mold, followed by photopolymerization under optimized conditions (Table 1). After polymerization, the gels were extracted, washed with methanol to remove unreacted monomers, neutralized using a triethylamine/methanol solution (1:9, *v*/*v*) to eliminate acidic impurities, and dried to constant weight. Swelling studies were performed by immersing the dried gels in the reaction solvent mixture, and solvent uptake was measured by determining the change in mass after 2 h and 24 h at room temperature and 40 °C (Supplementary Table S4).

The swelling behavior of the gels provided key insights into their catalytic performance. Swelling increased as the concentration of the catalytic monomer decreased, reaching equilibrium before eventual gel disintegration at 40 °C after 24 h. In contrast, all gels remained stable at room temperature, even after prolonged swelling. Optical microscopy images (Supplementary Figure S3) illustrate the morphological changes before and after swelling. Notably, composition A exhibited the highest stability at room temperature (Supplementary Figure S3(A3)), whereas compositions B, E, F, and G showed a morphological change due to excessive swelling. At 40 °C, only gel composition A remained stable after 2 h (Supplementary Figure S3(A5)), likely due to the higher content of non-polar methacrylate moieties.

**Figure 3:**
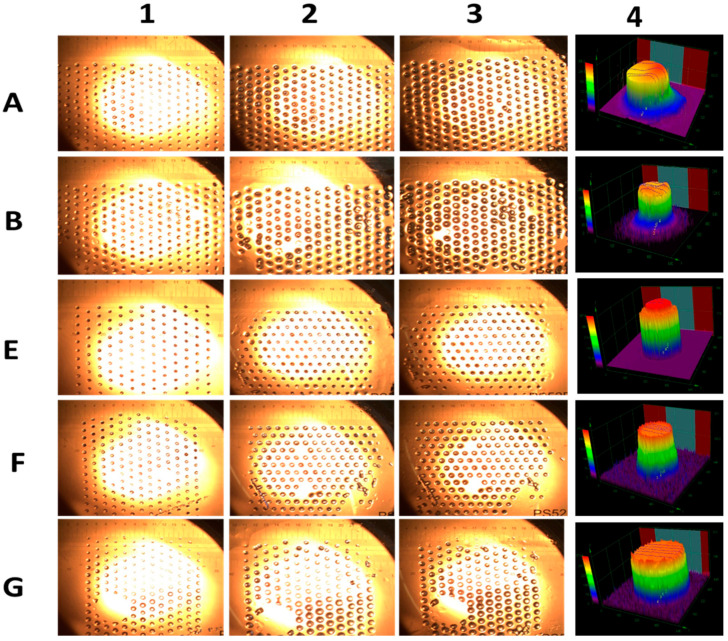
Optical microscopic images of gel dots **A**, **B**, **E**, **F,** and **G** before and after swelling. Here, **1**, **2**, and **3** indicate the swelling of the gels for 0 h, 2 h and 24 h, respectively. Figure right side **4** represents the laser microscope 3D images of all the dry cylindrical gels dots.

Additionally, gels containing 90% CAM (compositions A and E) exhibited lower swelling compared to B, F, and G, which contained a lower proportion of CAMs. More-over, stability increased with decreasing gel size, with polymeric gel dots showing enhanced resistance to disintegration [34]. These gel dots remained stable at 40 °C for 24 h in the reaction solvent, aided by their attachment to the surface (Figure 3). The size of the gel dots increased with swelling time, as seen in Figure 3(**2**,**3**), for all compositions. Furthermore, confocal microscopy 3D images (Figure 3(**4**)) confirmed the cylindrical morphology of the gel dots, with an average height of 250 ± 50 µm and a width of 350 ± 50 µm.

These findings highlight the crucial role of polymer network swelling in catalytic performance within MFRs, demonstrating that controlled gel stability and swelling behavior can significantly impact reaction efficiency.

**Scheme 2:**
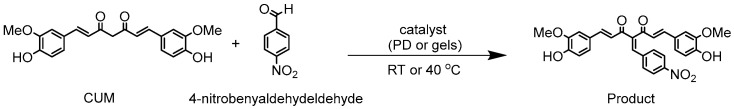
Synthesis of CUM derivatives via Knoevenagel reaction between 4-nitrobenzaldehyde and CUM in presence of homogeneous catalyst (PD) or in presence of heterogeneous catalyst (cylindrical gels **A**, **B**, **E**, **F**, and **G**) in batch reaction.

The reaction conditions for the synthesis of CUM derivatives were optimized by investigating the effects of solvent composition and temperature. In continuous MFR, solubility plays a crucial role in maintaining uninterrupted flow while preventing backpressure buildup or channel blockage. Thus, the solubility of CUM in various solvent mixtures was evaluated (Supplementary Table S2), with DMSO and methanol (7:3, *v*/*v*) providing complete solubility and enhanced swelling of the polymer networks. Consequently, all reactions were conducted in this solvent system. A batch reaction between 4-nitrobenzaldehyde and CUM was performed at different temperatures (room temperature and 40 °C) using varying molar ratios of 4-nitrobenzaldehyde to CUM (2:1 and 1:1) in the presence of a homogeneous catalyst (PD) at different concentrations (90%, 50%, and 20%; Scheme 2). The highest conversion (~88%) was achieved at 40 °C with a 2:1 molar ratio after 72 h (Supplementary Table S5). Conversion was determined via ^1^H NMR spectroscopy by analyzing the integrals of the aldehyde proton (δ = 10 ppm) and the product proton (δ = 8.1 ppm) (Supplementary Figures S4–S7).

Similarly, the Knoevenagel condensation between 4-nitrobenzaldehyde and CUM was carried out at different temperatures, under batch conditions, using heterogeneous catalysts based on cylindrical polymer networks (compositions A, B, E, F, and G; Scheme 2). Reaction progress was monitored at 24 h, 48 h, and 72 h via offline ^1^H NMR spectroscopy (Supplementary Table S6). No conversion was observed at room temperature, while at 40 °C, conversion increased with reaction time up to 72 h (Supplementary Figures S8–S12). Piperidine-4-yl methacrylate (**4**)-based catalysts (compositions A and B) exhibited distinct catalytic behaviors, with composition B achieving ~99% conversion, compared to ~34% for composition A, due to its higher swelling capacity. Similarly, piperidine-4-yl acrylate (**7**)-based catalysts (compositions E, F, and G) achieved conversions of 81%, 61%, and 34%, respectively (Supplementary Figure S13, reinforcing the correlation between swelling properties and catalytic efficiency. Notably, gel composition G, despite exhibiting high swelling, resulted in lower conversion (34%), suggesting that also a reduced catalyst concentration of 20% within the polymer network significantly influences catalytic activity.

Further batch reactions were conducted using various aldehydes with CUM in the presence of different catalysts over 72 h. Heterogeneous catalysts (compositions A and E) were compared to the homogeneous catalyst, PD (90%) (Table 2). Composition A exhibited lower conversion than composition E, which showed catalytic efficiency comparable to PD (~90%) (Supplementary Figure S14). This similarity suggests that the swelling properties of composition E enhanced catalyst accessibility, approximating the behavior of a homogeneous catalyst. Reactions with more electrophilic aldehydes (e.g., 4-nitrobenzaldehyde, 4-fluorobenzaldehyde, 4-chlorobenzaldehyde, 4-bromobenzaldehyde, and 3-chlorobenzaldehyde) yielded higher conversions than those with less reactive aldehydes, such as benzaldehyde and 4-methoxybenzaldehyde. Additionally, gel composition B achieved near-complete conversion (~100%) despite a lower catalyst concentration (50%). This trend further supports the role of swelling in increasing catalytic efficiency. Post-reaction, products were extracted with ethyl acetate and purified via column chromatography (30% ethyl acetate in *n*-hexane), with structural confirmation performed by ^1^H NMR spectroscopy (Supplementary Figures S15–S21).

To further optimize CUM derivative synthesis, reactions were performed in a continuous MFR using a hexagonal array of polymer gel dots (662 pieces) of compositions A, B, E, F, and G. The MFR setup included a syringe pump, a heated microfluidic reaction chamber, and a collection vial (Figure 1). Gel dots were placed within a PTFE reaction chamber, secured with parafilm tape, and mounted in an aluminum holder. PTFE capillary tubing (i.d. = 0.2 mm) connected the reactor to the syringe and collector vial.

Before reaction initiation, the gel dots were pre-swollen in DMSO/methanol (7:3, *v*/*v*) at 40 °C with a flow rate of 2 µL/min for 2 h. Swelling facilitated polymer chain expansion, improving catalyst accessibility. Subsequently, a reactant solution (2:1 molar ratio of aldehyde to CUM, 0.326 mmol and 0.163 mmol, respectively, in 4 mL of solvent) was introduced into the reactor at 0.5 µL/min and maintained at 40 °C. The reaction mixture was collected over 72 h after an initial residence time of 3.5 h. Conversion was quantified using offline ^1^H NMR spectroscopy by comparing the integrals of the product and aldehyde peaks.

The catalytic activity of gel dots (compositions A, B, E, F, and G) in the MFR was assessed via the reaction between 4-nitrobenzaldehyde and CUM at 40 °C over different time intervals (24 h, 48 h, and 72 h; Supplementary Table S7 and Figures S22–S25). Composition E achieved conversions of 28% (48 h) and 22% (72 h), while composition F showed improved performance, reaching 70% at 48 h and 72% at 72 h. Composition G, despite its high swelling capacity, displayed lower conversions (24%, 43%, and 32% at 24 h, 48 h, and 72 h, respectively), likely due to reduced catalyst concentration in the polymer network. In contrast, composition A exhibited lower catalytic activity, achieving 33% conversion at 24 h and 48% at 48 h, suggesting that limited swelling restricted reactant diffusion and catalyst accessibility [35]. Notably, composition B showed no conversion due to reactor clogging, highlighting the importance of balancing swelling properties with flow dynamics. Interestingly, this observation was reproducible and only seen for composition B. Gel B showed the best performance in batch reaction. Assuming the same for MFR, the solvent system might not be sufficient to solubilize the whole product. Finally, the stability of the gel dots might be so poor that gel dots suffering from disintegration caused the problem. Since optical inspection did not reveal any conspicuous features, further methods have to be utilized in the future.

Overall, reaction conversion in the MFR was significantly influenced by polymer swelling behavior and molecular complexity. Differences in conversion between batch and MFR reactions likely stem from variations in reaction conditions, catalyst distribution, and process limitations. Among the tested compositions, composition F demonstrated optimal performance (72% conversion at 72 h) due to its favorable swelling properties and balanced catalyst concentration, making it the preferred choice for CUM derivative synthesis in continuous MFR systems.

Finally, with the optimized gel composition, CUM derivatives were synthesized via the Knoevenagel reaction between CUM and various aldehydes at 40 °C for 72 h in a MFR. The reaction conversions are summarized in Table 3. Highly electrophilic aldehydes, such as 4-nitrobenzaldehyde, exhibited the highest conversion (72%), while less reactive aldehydes, such as 4-methoxybenzaldehyde, displayed significantly lower conversion (16%). Benzaldehyde derivatives yielded moderate conversion (47%). Halogenated benzaldehydes, including 4-chlorobenzaldehyde, 3-chlorobenzaldehyde, 4-bromobenzaldehyde, and 4-fluorobenzaldehyde, exhibited conversions of 27%, 31%, 22%, and 13%, respectively, potentially due to product–polymer interactions. MFR-based reactions generally exhibited lower conversions than batch reactions, likely due to the lower catalyst concentration (50%) in polymeric gel dots compared to cylindrical gels (90%) and homogeneous catalysts (PD, 90%). However, despite lower initial conversions, the turnover number of the catalyst in the MFR could increase over prolonged reaction times.

The catalytic efficiency of polymeric networks in MFRs is strongly dependent on swelling properties, catalyst distribution, and reactant diffusion. Optimizing these parameters enables competitive or superior catalytic performance compared to homogeneous systems, demonstrating the potential of polymeric catalysts for continuous-flow organic synthesis.

Reusability of gel bound catalysts has been proven in recent investigations [30,31,32]. Additionally, here, the reusability of the various gel compositions was determined by FTIR spectroscopy (Supplementary Figures S26–S30). Apart from small residues of the reactants, only weak bands were detected with regard to a structural change in the form of a protonation of the amino groups. However, since the band of the secondary amino group of the CAM is still clearly visible even after the reaction and no further structural changes were detected, the gels can be reused as heterogeneous catalysts.

## 3. Conclusions

The synthesis of bioactive compounds is gaining increasing significance in pharmaceutical research. In this study, we successfully demonstrated the synthesis of CUM derivatives via the Knoevenagel reaction, utilizing piperidine-based catalytic polymer networks in an MFR. Piperidine-based CAMs (**4**) and (**7**) were synthesized and polymerized into various compositions (A, B, E, F, and G) through photopolymerization, forming cylindrical gels and polymer gel dots. Optimization of reaction conditions was conducted with respect to solvent composition (best DMSO/methanol, 7:3 *v*/*v*) and temperature (best 40 °C). The polymer networks were subsequently employed as heterogeneous catalysts for the synthesis of CUM derivatives. Reaction conversions in both batch and continuous MFR systems were quantitatively assessed using offline ^1^H NMR spectroscopy. The findings indicated that reaction conversion increased with the swelling capacity of the polymer networks. Additionally, an increase in conversion with an increasing catalyst content of the gels could be demonstrated on the basis of compositions G-E. Notably, batch reactions employing gel composition B achieved a high conversion rate (99%) after 72 h. Unfortunately, the same composition in MFR showed clogging of the system. In contrast, in the continuous MFR system, gel dots of composition F exhibited superior catalytic performance, achieving a conversion of 72%, which was higher than the batch reaction conversion for the same composition (61%). Furthermore, the batch reactions required a greater quantity of catalyst compared to the continuous MFR process, in which the catalyst was utilized in a sustained manner. This suggests that the turnover number of the catalyst in the MFR system is significantly higher than in batch reactions, highlighting the efficiency of continuous-flow processing for catalytic transformations.

## 4. Materials and Methods

### 4.1. Materials

Piperidine (99%) and *tert*-butyl-4-hydroxypiperidine-1-carboxylate (95%) were purchased from ABCR GmbH (Karlsruhe, BW, Germany). Dimethylphenylphosphonite (98%), 3-(trichlorosilyl)propyl methacrylate (90%), triethylamine (99%), methyl methacrylate (98%) (MMA), *N*,*N*-methylenebis(acrylamide) (MBAM), and 4-methoxy benzaldehyde and 4-fluorobenzaldehyde 98% were purchased from Sigma Aldrich (Taufkirchen, BY, Germany). 2,4,6-Trimethylbenzoylchloride (>98%), acryloyl chloride (96%) with 400 ppm phenothiazine, 4-bromobenzaldehyde (99%), and methacryloyl chloride (97%) with ca. 400 ppm 4-methoxy-phenoland 2-butanone (99%) were purchased from Alfa Aesar (Haverhill, MA, USA). Dimethylsulfoxide (DMSO) (99.9%) was purchased from Grüssing GmbH (Filsum, NI, Germany). Curcumin (CUM) (99%), benzaldehyde (98%), aluminum oxide 90 active neutral, *N*,*N*-dimethylacrylamide (DMAM), ethylene glycol methacrylate (EGDMA), and 4N hydrogenchloridein1,4-dioxane were purchased from Tokyo Chemical Industries (TCI, Eschborn, HE, Germany. 4-Nitrobenzaldehyde (98%) was obtained from Merck (Darmstadt, HE, Germany). Di-*tert*-butyldicarbonate (>97%) and isobutyraldehyde (98%) were obtained from Thermo Scientific (Waltham, MA, USA). Deuterated chloroform (CDCl_3_) (99.8%) + Ag and deuterated dimethyl sulfoxide (DMSO-D_6_) were obtained from Deutero GmbH (Kastellaun, RP, Germany). Aqueous ammonia (25%), hydrogen peroxide (30%), 2-propanol (technical grade), methanol (technical grade), ethanol (technical grade), dichloromethane (DCM) (HPLC), citric acid, sodium chloride, aluminum oxide, and ethyl acetate were obtained from Stockmeier Chemie (Bielefeld, NW, Germany). Sodium hydrogen carbonate was procured from VWR International GmbH Chemicals (Langenfeld, NW, Germany). 3-Chlorobenzaldehyde (99%) was obtained from Acros Organics (Gleen, Belgium). Silica gel 60 was from Macherey-Nagel GmbH&Co. KG (Düren, NW, Germany). The photo-initiator, lithium phenyl-2,4,6-trimethylbenzoyl-phosphinate (LPTMBP), was synthesized according to reported procedure [36]. Methacryloyl chloride was distilled at 145 °C for synthesis of methacrylate derivative (**3**).

### 4.2. Characterizations

^1^H NMR (Nuclear Magnetic Resonance) spectra were recorded at the Avance500 of Bruker (Billerica, MA, USA) at 500 MHz or at the Ascent700 of Bruker (Billerica, MA, USA) at 700 MHz, and ^13^*C* NMR spectra were recorded at the Ascent700 at 176 MHz. The samples (5 mg) were taken in NMR tube and dissolved in 500–600 μL of deuterated solvents (CDCl_3_ or DMSO-d_6_). The prepared samples were scanned in the NMR spectrometer. Further, the obtained data were analyzed by software TopSpin 4.3.0, Bruker (Billerica, MA, USA). NMR signals were recorded with respect to the reference signal, tetramethyl silane (TMS). The electrospray ionization mass spectrometry (ESI-MS) measurements were performed on the device Synapt-2G HDMS of Waters (Milford, MA, USA) with a quadrupole time-of-flight (TOF) analyzer. All samples were dissolved in acetonitrile with a concentration of 1 mg/mL. A microscope from Hund (Wetzlar, HE, Germany) was used with a cold light source, FLQ 150 M. The images were taken with the camera iDS uEye UI146xLE-C and the software iDS uEYe cockpit 4.91.0 (iDS, Obersulm, BW, Germany). The measurements of the dimensions of the gels were made with a microscopic ruler of PYSER-SGI Ltd (Edenbridge, UK). The LEXT 3D Measuring Laser Confocal Microscope OLS4000 of Olympus (Shinjuku, Tokyo, Japan) was used for the recording of 3D images of gel dots and the measurement of their size with the acquisition parameters, scanning mode (XYZ fast scan), image size (1024 × 1024 pixels), and objective lens (MPLAPONLEXT20x). Attenuated total reflection (ATR) infrared spectra were recorded on a Bruker (Billerica, MA, USA) FTIR instrument (VERTEX 70) at room temperature. All spectra were measured with a resolution of 4 cm^−1^ in the 4500–350 cm^−1^ region.

### 4.3. Synthesis of CAM

(a) Synthesis of *tert*-butyl 4-(methacryloyloxy)piperidine-1-carboxylate (**3**):

The starting material of the piperidine derivative, *tert*-butyl-4-hydroxypiperidine-1-carboxylate (**1**) (10.013 g; 49.7 mmol), was taken in the 250 mL of two-necked round-bottom flask and the compound in 80 mL of dichloromethane (DCM). Triethylamine (8.27 mL; 59.6 mmol) was added to the reaction mixture and then stirred for 30 min at room temperature. Later, the reaction mixture was cooled to 0 °C using an ice bath, and then methacryloyl chloride (5.73 mL; 59.6 mmol) was added to the reaction mixture in a dropwise manner for 30 min in the presence of an inert gas atmosphere. After addition, the reaction mixture was stirred at room temperature for 18 h. After the completion of the reaction, the turbid reaction mixture was diluted with 80 mL DCM and filtered. Further, the filtrate was washed with water (160 mL) and then twice with saturated sodium hydrogen carbonate solution (160 mL × 2), ammonia solution (25%) (160 mL), and brine (160 mL). Finally, the organic phase was passed through a neutral alumina column to remove salts and then concentrated by a rotary evaporator to obtain colorless oil **3** (8.9 g).

Yield: 67% ^1^H-NMR (700 MHz, *CDCl*_3_): δ (ppm) = 1.47 (s, 9H), 1.67 (m, 2H), 1.87 (m, 2H), 1.95 (s, 3H), 3.32 (m, 2H), 3.66 (m, 2H), 5.01 (m, 1H), 5.56 (s, 1H), 6.12 (s, 1H). ^13^C-NMR (176 MHz, *DMSO-d*_6_): δ (ppm) = 18.6, 28.5, 31.0, 53.9, 70.1, 79.9, 126.0, 137.0, 155.0 and 167.2. ESI-MS (*m*/*z*): C_14_H_23_NO_4_Na^+^ [M–Na]^+^. Mass calculated: 292.1525 Da. Mass found: 292.1517 Da.

(b) Synthesis of piperidin-4-yl methacrylate (**4**):

The obtained compound **3** was placed in a 100 mL round-bottom flask, and 4 N HCl in 1,4-dioxane solution (50 mL) was added. Then, the reaction mixture was stirred at room temperature for 18 h. After completion of the reaction, the product was concentrated using a rotary evaporator. Furthermore, the product was dried under a high vacuum to yield colorless solid **4**.

Yield: 94% ^1^H-NMR (700 MHz, *DMSO-d*_6_): δ (ppm) = 1.57 (s, 3H), 2.10 (m, 2H), 2.29 (m, 2H), 3.28 (s, 4H), 5.13 (s, 1H), 5.65 (s, 1H), 6.13 (s, 1H), 9.74 (s, 2H). ^13^C-NMR (176 MHz, *DMSO-d*_6_): δ (ppm) = 18.6, 27.8, 40.8, 65.7, 127.0, 136.4 and 166.3. ESI-MS (*m*/*z*): C_9_H_16_NO_2_^+^ [M + H]^+^. Mass calculated: 170.1181 Da. Mass found: 170.1190 Da.

(c) Synthesis of tert-butyl 4-(acryloyloxy)piperidine-1-carboxylate (**6**):

Piperidine derivative, *tert*-butyl-4-hydroxypiperidine-1-carboxylate (**1**) (10.013 g; 49.7 mmol) was taken in the 250 mL two-necked round-bottom flask and the compound in 80 mL of dichloromethane (DCM). Triethylamine (8.27 mL; 59.6 mmol) was added to the reaction mixture and then stirred for 30 min at room temperature. Later, the reaction mixture was cooled to 0 °C using an ice bath, and then acryloyl chloride (4.86 mL, 59.6 mmol) was added to the reaction mixture in a dropwise manner for 30 min. After addition, the reaction mixture was stirred at room temperature for 18 h in the presence of an inert gas atmosphere. After the completion of the reaction, the turbid reaction mixture was diluted with 80 mL DCM and filtered. Further, the filtrate was washed with water (160 mL) and then twice with saturated sodium hydrogen carbonate solution (160 mL × 2), ammonia solution (25%) (160 mL), and brine (160 mL). Finally, the organic phase was passed through a neutral alumina column to remove salts and then concentrated by a rotary evaporator to obtain colorless oil **6** (9.71 g).

Yield: 70% ^1^H-NMR (700 MHz, CDCl_3_): δ (ppm) = 1.46 (s, 9H), 1.65 (m, 2H), 1.88 (m, 2H), 3.25 (m, 2H), 3.73 (m, 2H), 5.01 (m, 1H), 5.83 (dd, ^2^J_HH_ = 1.5 Hz, ^3^J_HH_ = 10.4 Hz, 1H), 6.11 (dd, ^3^J_HH,trans_ = 17.4 Hz, ^3^J_HH,cis_ = 10.4 Hz, 1H), 6.40 (dd, ^2^J_HH_ = 1.5 Hz, ^3^J_HH_ = 17.4 Hz, 1H). ^13^C-NMR (176 MHz, *DMSO-d*_6_): δ (ppm) = 28.5, 30.8, 53.4, 70.4, 80.1, 129.0, 131.1, 155.0 and 165.9. ESI-MS (*m*/*z*): C_13_H_21_NO_4_Na^+^ [M–Na]^+^. Mass calculated: 278.1368 Da. Mass found: 278.1378 Da.

(d) Synthesis of piperidin-4-yl acrylate (**7**):

The obtained compound, compound **6**, was placed in a 100 mL round-bottom flask, and 4 N HCl in 1,4-dioxane solution (50 mL) was added. Then, the reaction mixture was stirred at room temperature for 18 h. After completion of the reaction, the product was concentrated using a rotary evaporator. Furthermore, the product was dried under a high vacuum to yield colorless solid **7**.

Yield: 96% ^1^H-NMR (700 MHz, *DMSO-d*_6_): δ (ppm) = 2.09 (m, 2H), 2.27 (m, 2H), 3.29 (m, 4H), 5.14 (s, 1H), 5.91 (d, ^3^J_HH,cis_ = 10.8 Hz, 1H), 6.12 (dd, ^3^J_HH,trans_ = 17.5 Hz, ^3^J_HH,cis_ = 11.1 Hz, 1H), 6.44 (d, ^3^J_HH,trans_ = 17.5 Hz, 1H), 9.72 (s, 2H). ^13^C-NMR (176 MHz, *DMSO-d*_6_): δ (ppm) = 27.4, 40.8, 65.7, 128.2, 132.1 and 165.3. ESI-MS (*m*/*z*): C_8_H_14_NO_2_^+^ [M + H]^+^. Mass calculated: 156.1025 Da. Mass found: 156.1036 Da.

### 4.4. Optimization of Photopolymerization Parameters for Different Polymer Network Compositions

Polymeric networks were fabricated with various compositions (A, B, C, D, E, F, and G) of CAM (**4** or **7**) complimentary monomer (MMA or DMAA) and crosslinker (EGDMA or MBAM) by photopolymerization, and the polymerization conditions were optimized, as given in Supplementary Table S1. The monomer solution (15 mmol) in water was prepared in a glass vial, and photo-initiator (17.5 mg/mL) was added to the monomeric solution in dark conditions and then stirred for 2 h. Further, the prepared solution was taken in a Pasteur pipette and irradiated using the instrument Omnicure^®^ S1500 UV-lamp from Lumen Dynamics (Mississauga, ON, Canada) for the respective time and intensity given in the Supplementary Table S1. After the optimal polymerization conditions, the resulting gels were washed in methanol overnight and neutralized with methanol/triethylamine (9:1, *v*/*v*) for 1 h. After neutralization, the gels were washed twice with methanol for 0.5 h. The obtained gels were dried at room temperature until mass consistency and were then ready for swelling studies.

A: FTIR: $\tilde{v}$ (cm^−1^) = 3389 ν(N-H), 2951 ν(CH_3_)_s_, 2806 ν(CH_3_)_as_, 2727 ν(CH_2_), 1716 ν(C=O), 1633 δ(N-H), 1454 δ(C-H)_as_, 1242 δ(C-H)_s_, 1155 ν(C-N), 1026 ν(O=C-O-C), 962 ν(C-O-C).

B: FTIR: $\tilde{v}$ (cm^−1^) = 3389 ν(N-H), 2951 ν(CH_3_)_s_, 2829 ν(CH_3_)_as_, 2731 ν(CH_2_), 1718 ν(C=O), 1634 δ(N-H), 1448 δ(C-H)_as_, 1242 δ(C-H)_s_, 1149 ν(C-N), 1027 ν(O=C-O-C), 962 ν(C-O-C).

E: FTIR: $\tilde{v}$ (cm^−1^) = 3419 ν(N-H), 2997 ν(CH_3_)_s_, 2916 ν(N-CH_3_), 2816 ν(CH_3_)_as_, 2725 ν(CH_2_), 1728 ν(C=O), 1633 δ(N-H), 1437 δ(C-H)_as_, 1406 ν(C-N), 1256 δ(C-H)_s_, 1161 ν(C-N), 1016 ν(O=C-O-C), 953 ν(C-O-C).

F: FTIR: $\tilde{v}$(cm^−1^) = 3385 ν(N-H), 2935 ν(CH_3_)_s_, 2856 ν(CH_3_)_as_, 2728 ν(CH_2_), 1724 ν(C=O), 1614 δ(N-H), 1454 δ(C-H)_as_, 1400 ν(C-N), 1257 δ(C-H)_s_, 1140 ν(C-N), 1034 ν(O=C-O-C), 960 ν(C-O-C).

G: FTIR: $\tilde{v}$ (cm^−1^) = 3391 ν(N-H), 2933 ν(CH_3_)_s_, 2829 ν(CH_2_), 1722 ν(C=O), 1614 δ(N-H), 1454 δ(C-H)_as_, 1142 ν(C-N), 1034 ν(O=C-O-C), 951 ν(C-O-C).

### 4.5. Swelling Studies of the Polymer Networks

The swelling properties of the polymer networks were performed in the reaction solvent mixture, DMSO/methanol (7:3, *v*/*v*), at room temperature and 40 °C. The known weights of cylindrical gels A, B, E, F, and G were taken in the glass vial and swollen in the reaction solvent mixture, DMSO/methanol (7:3, *v*/*v*), at room temperature and 40 °C. The swelling properties of the gels were evaluated at different time intervals, 2 h and 24 h. After the respective time of swelling, the gels were taken out from the glass vial, and the gels (before and after swelling) were measured to determine the solvent uptake. The percentage of the solvent uptake (*W_M_*) was calculated using Equation (1). The swelling studies were performed for all samples in duplicate.

(1) $$W_{M}=\frac{w_{2}-w_{1}}{w_{1}}\cdot 100\%$$

where

*W*_2_ = weight of the swollen gel (mg);
*W*_1_ = weight of the dry gel (mg).

Moreover, images of the gels (before and after swelling) were captured using optical microscopy, Hund Wetzlar, with a cold light source, FLQ 150 M. The images were taken with the camera iDS uEye UI146xLE-C and the software iDS uEYe cockpit 4.91.0.

### 4.6. Fabrication of Polymer Gel Dots Using Various Compositions of Monomer Solution

Fabrication of polymer gel dots was performed on the surface of microscopic glass slides. Initially, the modification of glass slides (7.6 cm × 2.6 cm) was performed as per the reported procedure [33]. Briefly, the microscopic glass slides (10 numbers) were washed with water, ethanol, and isopropanol in an ultrasonic bath for 10 min with each solvent. Afterward, the surface of glass slides was oxidized by using a mixture of 200 mL de-ionized water, 40 mL of ammonia solution (25%), and 40 mL of hydrogen peroxide solution (35%) for 10 min at 70 °C under sonication. After oxidation, the glass slides dried using a nitrogen gas equipped with nitrogen gas pistol and stored in the desiccation. Further, the modification of the hydroxyl groups on the surface of glass slide using 150 μL of 3-(trichlorisilyl)propyl methacrylate by vapor adsorption in the desiccator under vacuum of at least 50 mbar was applied for 2 h. These modified microscopic glass slides were used for the preparation of polymer gel dots. Initially, various compositions, A, B, E, F, and G, of monomer solutions (15 mmol) in water were prepared with photo-initiator (LPTMBP) (17.5 mg/mL) and stirred for 2 h in the dark condition. Further, the prepared solution (850 μL) was taken in an incubator chamber gasket and spread evenly, and then the solution was covered by modified microscopic glass (without disturbing the surface of the glass slide). Then, on top of the photomask, the containing 665 dots were placed. The sandwich assembly was placed under the UV light for photopolymerization. The parameters of the photopolymerization for the respective gel compositions, A, B, E, F, and G, are given in Table 1. After irradiation, the gel dots were carefully taken out from the gasket and washed with methanol, and then they were neutralized with methanol/triethylamine (9:1. *v*/*v*) solution for 1 h and washed again twice with methanol for 0.5 h. Finally, the obtained gel dots were dried at room temperature for one day and stored in a dry place until further use. The size and shape of the gel dots A, B, E, F, and G were analyzed with the Laser Confocal Microscope OLS4000 LEXT of Olympus.

### 4.7. General Procedure for Synthesis of CUM Derivatives in Batch Reactions Using Different Catalysts

CUM (0.082 mmol, 1 eq.) and respective aldehyde (0.168 mmol, 2 eq. or 0.082 mmol, 1 eq.) were taken in a 10 mL of sample vial and dissolved in 2 mL of the reaction solvent mixture, DMSO/methanol (7:3, *v*/*v*). Further, the respective catalyst (homogeneous catalyst, piperidine (PD), or heterogeneous catalyst, cylindrical gel (approximately 20 mg of catalyst and was used once during the experiment)) was added to the reaction mixture and stirred at respective temperatures (room temperature and 40 °C) for the respective reaction time (24 h, 48 h, and 72 h). After completion of the reaction, conversion was determined using offline ^1^H NMR spectroscopy. A mixture of 100 µL of reaction mixture and 500 µL DMSO-d6 was taken in the NMR tube, and we scanned the sample using an NMR spectrometer. Determination of conversion was performed using the integral of aldehyde peak and the respective product peak. Further, the product was isolated by the solvent extraction method. After completion of the reaction, the reaction mixture was diluted with 5 mL of water and extracted with 3 × 10 mL of ethyl acetate. The obtained organic phase was concentrated by using rotary evaporation. The crude product was further purified using a silica column with a solvent mixture of ethyl acetate and *n*-hexane. The solvent ratio was increased from 5:95 with a gradient to 30:70. The product was concentrated using rotary evaporation and dried under high vacuum. The products were obtained as yellow solids.

*Synthesis of (1E,6E)-1,7-bis(4-hydroxy-3-methoxyphenyl)-4-(4-nitrobenzylidene)-hepta-1,6-diene-3,5-dione:*

^1^H-NMR (500 MHz, DMSO-d6): δ (ppm) = 3.79 (s, 3H), 3.86 (s, 3H), 6.82 (m, 2H), 6.88 (d, ^3^J_HH,trans_ = 24.2 Hz, 1H), 7.09 (dd, ^3^J_HH_ = 8.3 Hz, ^4^J_HH_ = 1.9 Hz, 1H), 7.28 (d, ^4^J_HH_ = 1.9 Hz, 1H), 7.30 (dd, ^3^J_HH_ = 8.2 Hz, ^4^J_HH_ = 1.9 Hz, 1H), 7.41 (d, ^3^J_HH,trans_ = 16.1 Hz, 1H), 7.44 (d, ^4^J_HH_ = 2.1 Hz, 1H), 7.54 (d, ^3^J_HH,trans_ = 15.4 Hz, 1H), 7.66 (d, ^3^J_HH,trans_ = 15.2 Hz, 1H), 7.77 (d, ^3^J_HH_ = 8.9 Hz, 2H), 8.12 (s, 1H), 8.26 (d, ^3^J_HH_ = 9.0 Hz, 2H), 9.61 (s, 2H). ESI-MS (*m*/*z*): C_28_H_22_NO_8_^+^ [M]^+^. Mass calculated: 500.1345 Da. Mass found: 500.1348 Da.

*Synthesis of (1E,6E)-4-(4-fluorobenzylidene)-1,7-bis(4-hydroxy-3-methoxyphenyl)-hepta-1,6-diene-3,5-dione:*

^1^H-NMR (500 MHz, DMSO-d6): δ (ppm) = 3.79 (s, 3H), 3.87 (s, 3H), 6.79 (d, ^3^J_HH_ = 8.0 Hz, 1H), 6.86 (d, ^3^J_HH_ = 7.9 Hz, 1H), 6.88 (d, ^3^J_HH,trans_ = 15.6 Hz, 1H), 7.09 (dd, ^3^J_HH_ = 8.5 Hz, ^4^J_HH_ = 2.0 Hz, 1H), 7.29 (dd, ^3^J_HH_ = 7.9 Hz, ^4^J_HH_ = 2.2 Hz, 1H), 7.31 (d, ^4^J_HH_ = 2.0 Hz, 1H), 7.41 (d, ^3^J_HH,trans_ = 15.9 Hz, 1H), 7.44 (d, ^4^J_HH_ = 2.1 Hz, 1H), 7.54 (d, ^3^J_HH,trans_ = 15.6 Hz, 1H), 7.66 (d, ^3^J_HH,trans_ = 15.5 Hz, 1H), 7.77 (d, ^3^J_HH_ = 8.8 Hz, 2H), 8.0 (s, 1H), 8.26 (d, ^3^J_HH_ = 9.0, 2H), 9.73 (s, 2H). ESI-MS (*m*/*z*): C_28_H_22_O_6_F [M]^+^. Mass calculated: 473.1400 Da. Mass found: 473.1404 Da.

*Synthesis of (1E,6E)-4-(4-bromobenzylidene)-1,7-bis(4-hydroxy-3-methoxyphenyl)-hepta-1,6-diene-3,5-dione:*

^1^H-NMR (500 MHz, DMSO-d6): δ (ppm) = 3.79 (s, 3H), 3.85 (s, 3H), 6.74 (d, ^3^J_HH,trans_ = 16.4 Hz, 2H), 6.77 (d, ^3^J_HH_ = 8.8 Hz, 1H), 6.86 (d, ^3^J_HH_ = 8.0 Hz, 1H), 7.08 (dd, ^3^J_HH_ = 8.4 Hz, ^4^J_HH_ = 1.8 Hz, 1H), 7.27 (m, 2H), 7.41 (d, ^4^J_HH_ = 2.0 Hz, 1H), 7.47 (d, ^3^J_HH_ = 8.6 Hz, 2H), 7.58 (d, ^3^J_HH,trans_ = 14.7 Hz, 2H), 7.62 (m, 2H), 7.98 (s, 1H), 9.75 (s, 2H). ESI-MS (*m*/*z*): C_28_H_22_O_6_Br [M − H]^+^. Mass calculated: 533.0600 Da. Mass found: 533.0593 Da.

*Synthesis of (1E,6E)-4-(4-chlorobenzylidene)-1,7-bis(4-hydroxy-3-methoxyphenyl)-hepta-1,6-diene-3,5-dione:*

^1^H-NMR (500 MHz, DMSO-d6): δ (ppm) = 3.79 (s, 3H), 3.85 (s, 3H), 6.77 (d, ^3^J_HH_ = 8.0 Hz, 1H), 6.85 (d, ^3^J_HH_ = 7.7 Hz, 1H), 6.87 (d, ^3^J_HH,trans_ = 16.0 Hz, 1H), 7.08 (dd, ^3^J_HH_ = 8.3 Hz, ^4^J_HH_ = 2.0 Hz, 1H), 7,27 (m, 2H, H-3), 7.38 (d, ^3^J_HH,trans_ = 16.0 Hz, 1H), 7.40 (d, ^3^J_HH,trans_ = 15.3, 1H), 7.41 (d, ^4^J_HH_ = 2.1 Hz, 1H), 7.48 (d, ^3^J_HH_ = 8.6 Hz, 2H), 7.54 (d, ^3^J_HH_ = 10,1 Hz, 2H), 7.61 (d, ^3^J_HH,trans_ = 15.0, 1H), 8.00 (s, 1H), 9.75 (s, 2H). ESI-MS (*m*/*z*): C_28_H_22_O_6_Cl [M − H]^+^. Mass calculated: 489.1105 Da. Mass found: 489.1106 Da.

*Synthesis of (1E,6E)-4-(3-chlorobenzylidene)-1,7-bis(4-hydroxy-3-methoxyphenyl)-hepta-1,6-diene-3,5-dione:*

^1^H-NMR (500 MHz, DMSO-d6): δ (ppm) = 3.80 (s, 3H), 3.86 (s, 3H), 6.72 (d, ^3^J_HH_ = 7.5 Hz, 1H), 6.78 (d, ^3^J_HH_ = 8.0 Hz, 1H), 6.86 (d, ^3^J_HH,trans_ = 11.5 Hz, 1H), 6.88 (d, ^3^J_HH,trans_ = 18.1 Hz, 1H), 7.10 (dd, ^3^J_HH_ = 8.2 Hz, ^4^J_HH_ = 1.9 Hz, 1H), 7.29 (m, 2H), 7.44 (m, 3H), 7.48 (m, 1H), 7.63 (m, 3H), 8.01 (s, 1H), 9.70 (s, 2H). ESI-MS (*m*/*z*): C_28_H_22_O_6_Cl [M − H]^+^. Mass calculated: 489.1105 Da. Mass found: 489.1108 Da.

*Synthesis of (1E,6E)-4-benzylidene-1,7-bis(4-hydroxy-3-methoxyphenyl)hepta-1,6-diene-3,5-dione:*

^1^H-NMR (500 MHz, DMSO-d6): δ (ppm) = 3.79 (s, 3H), 3.86 (s, 3H), 6.77 (d, ^3^J_HH_ = 8.9 Hz, 1H), 6.85 (d, ^3^J_HH_ = 8.9 Hz, 1H), 6.88 (d, ^3^J_HH,trans_ = 17.1 Hz, 1H), 7.08 (dd, ^3^J_HH_ = 8.5 Hz, ^4^J_HH_ = 1.4 Hz, 1H), 7.28 (m, 2H), 7.38 (d, ^3^J_HH,trans_ = 16.4 Hz, 1H), 7.43 (d, ^4^J_HH_ = 1.9 Hz, 1H), 7.58 (m, 4H), 7.73 (tt, ^3^J_HH_ = 7.4 Hz, ^4^J_HH_ = 1.3 Hz, 1H), 7.93 (dd, ^3^J_HH_ = 8.0 Hz, ^4^J_HH_ = 1.4 Hz, 2H), 8.02 (s, 1H), 9.68 (s, 2H). ESI-MS (*m*/*z*): C_28_H_23_O_6_ [M − H]^+^. Mass calculated: 455.1495 Da. Mass found: 455.1498 Da.

*Synthesis of (1E,6E)-1,7-bis(4-hydroxy-3-methoxyphenyl)-4-(4-methoxybenzylidene)hepta-1,6-diene-3,5-dione:*

^1^H-NMR (500 MHz, *CDCl*_3_): δ (ppm) = 3.78 (s, 3H), 3.80 (s, 3H), 3.86 (s, 3H), 6.79 (d, ^3^J_HH_ = 8.4 Hz, 1H), 6.86 (d, ^3^J_HH_ = 8.8 Hz, 1H), 6.89 (d, ^3^J_HH,trans_ = 13.1 Hz, 1H), 6.99 (d, ^3^J_HH_ = 8.8 Hz, 2H), 7.09 (dd, ^3^J_HH_ = 8.4 Hz, ^4^J_HH_ = 2.1 Hz, 1H), 7.16 (d, ^3^J_HH_ = 8.4 Hz, 2H), 7.27 (m, 2H), 7.38 (d, ^3^J_HH,trans_ = 15.9 Hz, 1H), 7.41 (d, ^4^J_HH_ = 1.9 Hz, 1H), 7.51 (d, ^3^J_HH,trans_ = 10.6 Hz, 1H), 7.58 (d, ^3^J_HH,trans_ = 15.2 Hz, 1H), 7.98 (s, 1H), 9.68 (s, 2H). ESI-MS (*m*/*z*): C_29_H_25_O_7_ [M − H]^+^. Mass calculated: 485.1600 Da. Mass found: 485.1600 Da.

### 4.8. Assembly of Microfluidic Reactor and Microfluidic Reactions

The microfluidic reactor was assembled according to the previous procedure [37]. Briefly, the microfluidic reactor system mainly consists of three components: a syringe pump, reactor, and colleting vial. The flow rate of the reactant solution was controlled using a “Legato 200” syringe pump (KD Scientific), which was equipped with syringe and a “Hamilton 1000 series” as a reservoir. Further, the reactor was assembled with a reaction chamber (hexagonal shaped) with an imprinted PTFE layer, and it was covered with a microscopic glass slide containing hexagonal-shaped polymeric gel dots (662 number). The microfluidic reactor was shielded with parafilm and fixed with self-made alumina holders by screws with torque of 0.8 Nm. The inlet from the syringe and the outlet to the collecting vial of the reactor were connected with the PTFE capillary tubes (iD = 0.2 mm) from Fisher Scientific. Further, the reaction chamber was placed on the thermal plate made of aluminum. After assembly of the reactor, microfluidic reactions were performed for synthesis of CUM derivatives at 40 °C. Initially, the reaction chamber was heated at 40 °C, and then the polymeric gel dots in the MFR were pre-swollen in the pure reaction solvent mixture, DMSO/methanol (7:3, *v*/*v*), for 2 h, with a flow rate of 2.0 µL/min. After the pre-swelling process, the syringe was loaded with reactant solution (CUM (60.0 mg; 0.163 mmol; 1eq.) and aldehyde (0.326 mmol, 2. eq.) dissolved in 4 mL of DMSO/methanol mixture (7:3, *v*/*v*)), and the flow rate was adjusted to 0.5 µL/min at the same temperature. The product was collected with a constant flow rate for 72 h after the initial residence time (3.45 h) in intervals of 24 h, and the conversion was determined by offline ^1^H NMR spectroscopy. The conversion was calculated by dividing the integral of product peak by the sum of integral of product peak and the integral of the aldehyde peak. This term had to be multiplied by 2 for 2 eq. of aldehyde with respect to CUM, 1 eq. Similarly, all the MFR reactions for synthesis of CUM derivatives were performed, and the spectral data are as given in Supplementary Figure S31.

### 4.9. Reusability of Polymer Gels

To analyze the reusability of the polymer gels, they were removed from the reaction mixture after the heterogeneous catalysis of a reaction of CUM with 4-nitrobenzaldehyde. After washing with a DMSO/methanol solution (7:3), the structure of the gels was examined using ATR-FTIR spectroscopy, since it had been shown in previous studies that this method can be used to determine the reusability of the gels [33]. The FTIR spectra are given in Supplementary Figures S27–S31.

A: FTIR: $\tilde{v}$ (cm^−1^) = 3419 ν(N-H), 2999 ν(N-H_2_), 2916 ν(CH_3_)_s_, 2825 ν(CH_3_)_as_, 1720 ν(C=O), 1605 δ(N-H), 1520 ν(R-NO_2_), 1437 δ(C-H)_as_, 1406 ν(C=C), 1344 ν(C=C), 1313 ν(C=C), 1252 δ(C-H)_s_, 1155 ν(C-N), 1016 ν(O=C-O-C), 953 ν(C-O-C).

B: FTIR: $\tilde{v}$ (cm^−1^) = 3425 ν(N-H), 3268 ν(N-H_2_), 2916 ν(CH_3_)_s_, 2822 ν(CH_3_)_as_, 2733 ν(CH_2_), 1726 ν(C=O), 1632 δ(N-H), 1518 ν(R-NO_2_), 1437 δ(C-H)_as_, 1404 ν(C=C), 1346 ν(C=C), 1257 ν(C=C), 1163 ν(C-N), 1018 ν(O=C-O-C), 953 ν(C-O-C).

E: FTIR: $\tilde{v}$ (cm^−1^) = 3385 ν(N-H), 3244 ν(N-H_2_), 2947 ν(CH_3_)_s_, 2818 ν(CH_3_)_as_, 2727 ν(CH_2_), 1724 ν(C=O), 1621 δ(N-H), 1560 ν(R-NO_2_), 1448 δ(C-H)_as_, 1394 ν(C=C), 1375 ν(C=C), 1254 δ(C-H)_s_, 1161 ν(C-N), 1026 ν(O=C-O-C), 960 ν(C-O-C).

F: FTIR: $\tilde{v}$ (cm^−1^) = 3402 ν(N-H), 3275 ν(N-H_2_), 3000 ν(CH_3_), 2918 ν(CH_2_), 1722 ν(C=O), 1634 δ(N-H), 1520 ν(R-NO_2_), 1437 δ(C-H)_as_, 1346 ν(C=C), 1242 δ(C-H)_s_, 1153 ν(C-N), 1016 ν(O=C-O-C), 953 ν(C-O-C).

G: FTIR: $\tilde{v}$ (cm^−1^) = 3431 ν(N-H), 3281 ν(N-H_2_), 2999 ν(CH_3_)_s_, 2918 ν(CH_2_), 1724 ν(C=O), 1628 δ(N-H), 1435 δ(C-H)_as_, 1402 ν(C=C), 1347 ν(C=C), 1313 ν(C=C), 1255 δ(C-H)_s_, 1140 ν(C-N), 1020 ν(O=C-O-C), 953 ν(C-O-C).

## Figures and Tables

**Table 1:** Different compositions of piperidin-4-yl methacrylate and acrylate gels and polymerization parameters for photo patterning.

Composition Code	Catalytic Monomer	Complementary Monomer	Crosslinker	Solvent	Concentration (15 mmol in x mL)	UV Irradiation Time (s)	UV Intensity (W)
A	**4** (90%)	MMA (5%)	EGDMA (5%)	H_2_O	0.8	10	0.42
B	**4** (50%)	MMA (45%)	EGDMA (5%)	H_2_O	0.6	35	0.84
C *	**4** (30%)	MMA (65%)	EGDMA (5%)	H_2_O	0.6	140	1.28
D *	**4** (20%)	MMA (75%)	EGDMA (5%)	H_2_O	0.6	140	1.28
E	**7** (90%)	DMAA (5%)	MBAM (5%)	H_2_O	0.8	16	0.42
F	**7** (50%)	DMAA (45%)	MBAM (5%)	H_2_O	0.6	11	0.42
G	**7** (20%)	DMAA (75%)	MBAM (5%)	H_2_O	0.6	5	0.42

* No gel formation.

**Table 2:** Synthesis of different CUM derivatives in batch reactions using various catalysts, PD (homogeneous catalyst, 90% piperidine), **A** (heterogeneous catalyst, cylindrical gel composition **A**), and **E** (heterogeneous catalyst, cylindrical gel composition **E**).

Entry No.	Aldehyde	Product	Conversion [%] with Catalyst		
			PD	A	E
1	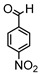	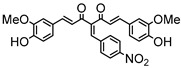	89	34	82
2	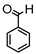	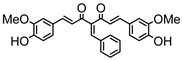	59	19	70
3	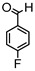	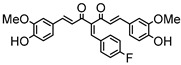	49	21	70
4	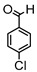	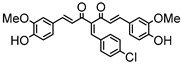	53	9	61
5	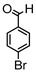	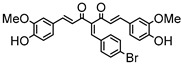	52	3	68
6	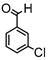	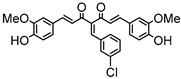	71	3	63
7	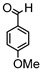	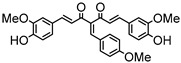	35	11	29

**Table 3:** Synthesis of different CUM derivatives in MFR using gel dot composition F.

Entry No.	Aldehyde	Product	Conversion [%] with Catalyst F
1	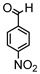	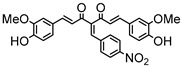	72
2	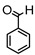	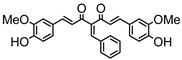	47
3	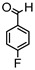	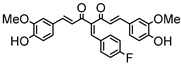	13
4	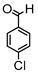	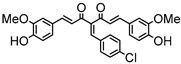	27
5	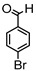	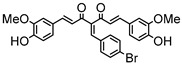	22
6	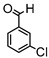	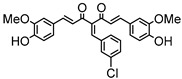	31
7	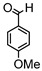	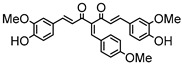	16

## Data Availability

The original contributions presented in this study are included in the article/Supplementary Material. Further inquiries can be directed to the corresponding author.

## Supplementary Materials

The following supporting information can be downloaded at https://www.mdpi.com/article/10.3390/gels11040278/s1. Figure S1: 1H NMR spectrum of 4-(methacryloyloxy)piperidin-1-ium chloride; Figure S2: 1H NMR spectrum of 4-(acryloyloxy)piperidin-1-ium chloride; Figure S3: Optical microscopic images of cylindrical gels; Figure S4: 1H NMR spectra of entry number 1 in Table S5; Figure S5: 1H NMR spectra of entry number 2 in Table S5; Figure S6: 1H NMR spectra of entry number 3 in Table S5; Figure S7: 1H NMR spectra of entry number 4 in Table S5; Figure S8: 1H NMR spectra of entry number 2 in Table S6; Figure S9: 1H NMR spectra of entry number 3 in Table S6; Figure S10: 1H NMR spectra of entry number 4 in Table S6; Figure S11: 1H NMR spectra of entry number 5 in Table S6; Figure S12: 1H NMR spectra of entry number 6 in Table S6; Figure S13: Conversions at different times of batch reactions of curcumin (CUM) with 4-nitrobenzaldehyde catalysed with gels of the compositions A, B, E, F, and G; Figure S14: Graphical comparison of conversion in batch reactions, which were homogenously catalysed with piperidine (PD) and heterogeneously catalysed with cylindrical gels; Figure S15: 1H NMR spectrum of (1E,6E)-1,7-bis(4-hydroxy-3-methoxyphenyl)-4-(4-nitrobenzylidene)hepta-1,6-diene-3,5-dione; Figure S16: 1H NMR spectrum of (1E,6E)-4-benzylidene-1,7-bis(4-hydroxy-3-methoxyphenyl)hepta-1,6-diene-3,5-dione; Figure S17: 1H NMR spectrum of (1E,6E)-4-(4-fluorobenzylidene)-1,7-bis(4-hydroxy-3-methoxyphenyl)hepta-1,6-diene-3,5-dione; Figure S18: 1H NMR spectrum of (1E,6E)-4-(4-chlorobenzylidene)-1,7-bis(4-hydroxy-3-methoxyphenyl)hepta-1,6-diene-3,5-dione; Figure S19: 1H NMR spectrum of (1E,6E)-4-(4-bromobenzylidene)-1,7-bis(4-hydroxy-3-methoxyphenyl)hepta-1,6-diene-3,5-dione; Figure S20: 1H NMR spectrum of (1E,6E)-4-(3-chlorobenzylidene)-1,7-bis(4-hydroxy-3-methoxyphenyl)hepta-1,6-diene-3,5-dione; Figure S21: 1H NMR spectrum of (1E,6E)-1,7-bis(4-hydroxy-3-methoxyphenyl)-4-(4-methoxybenzylidene)hepta-1,6-diene-3,5-dione;Figure S22: 1H NMR spectra of entry number 1 in Table S7; Figure S23: 1H NMR spectra of entry number 2 in Table S7; Figure S24: 1H NMR spectra of entry number 3 in Table S7; Figure S25: 1H NMR spectra of entry number 4 in Table S7; Figure S26: FTIR spectra of gels of composition A before and after catalysis of reactions of CUM with 4-nitrobenzaldehyde; Figure S27: FTIR spectra of gels of composition B before and after catalysis of reactions of CUM with 4-nitrobenzaldehyde; Figure S28: FTIR spectra of gels of composition E before and after catalysis of reactions of CUM with 4-nitrobenzaldehyde; Figure S29: FTIR spectra of gels of composition F before and after catalysis of reactions of CUM with 4-nitrobenzaldehyde; Figure S30: FTIR spectra of gels of composition G before and after catalysis of reactions of CUM with 4-nitrobenzaldehyde; Figure S31: 1H NMR spectra of curcumin derivatives synthesized in the MFR using gel dots composition F for 72 h; Table S1: Different compositions of piperidin-4yl methacrylate and acrylate gels and polymerization parameters for bulk gels; Table S2: Solubility tests of curcumin at room temperature and at 40 °C; Table S3: Swelling behaviour of gels in different solvents; Table S4: Percentages of solvent uptake after 2 h and 24 h of gels of composition A and B; Table S5: Conversion of batch reaction of 4-nitrobenzaldehyde with CUM and different contents of homogeneous catalyst PD; Table S6: Conversion of heterogeneously catalysed batch reactions of CUM with 4-nitrobenzaldehyde catalysed by cylindrical gels of compositions A, B, E, F, and G; Table S7: Conversion of microflow reactions of CUM with 4-nitrobenzaldehyde catalysed by 662 gel dots of compositions A, B, E, F, and G.
