# Supplementary material for: Synthesis of Curcumin Derivatives via Knoevenagel Reaction Within a Continuously Driven Microfluidic Reactor Using Polymeric Networks Containing Piperidine as a Catalyst
Source: Gels. 2025 Apr 8;11(4):278. doi: 10.3390/gels11040278 (PMC12026977; doi:10.3390/gels11040278)
Supplement: Supplementary file 1 [file gels-11-00278-s001.zip › gels-3531055-supplementary.pdf]

Supporting Information

Synthesis of Curcumin Derivatives via Knoevenagel Reaction Within a Continuously Driven Microfluidic Reactor Using Polymeric Networks Containing Piperidine as a Catalyst

Naresh Killi, Katja Rumpke and Dirk Kuckling *

Department of Chemistry, Faculty of Science, Paderborn University, Warburger Str. 100, 33098 Paderborn, Germany; naresh.killi@uni-paderborn.de (N.K.); krumpke@mail.uni-paderborn.de (K.R.)
* Correspondence: dirk.kuckling@uni-paderborn.de

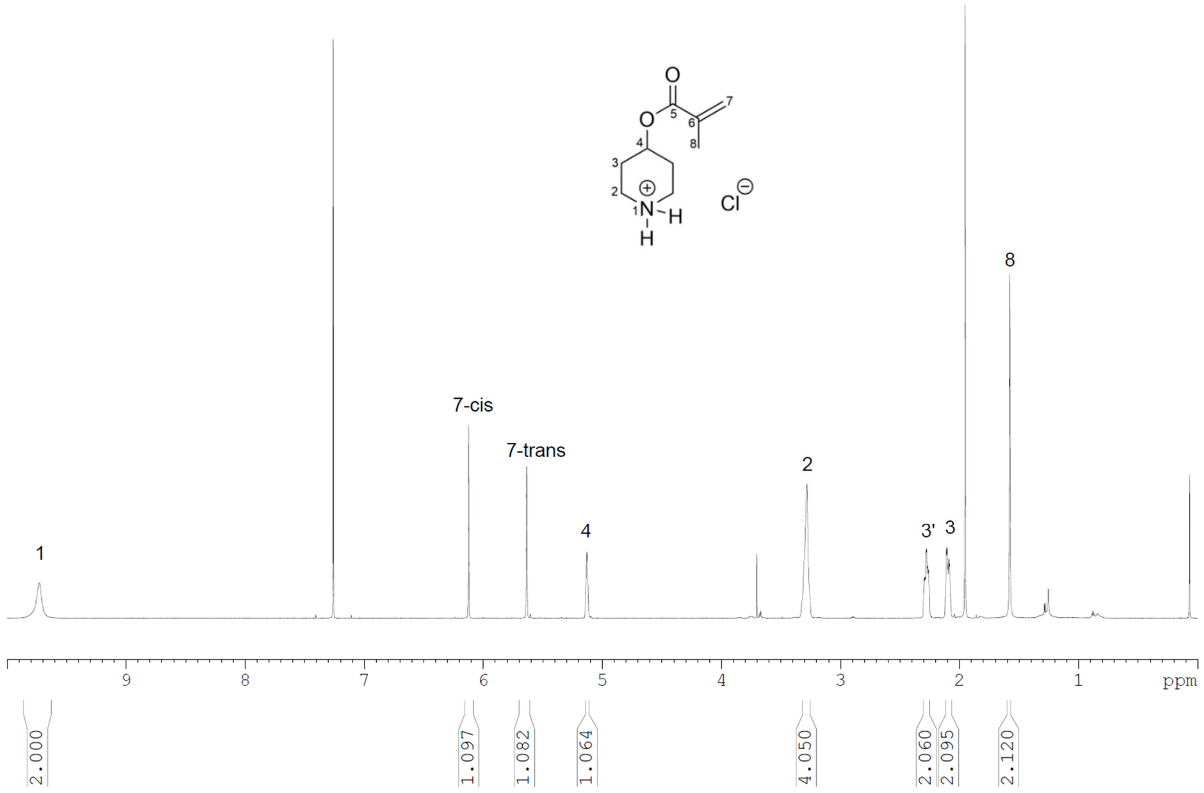

Figure S1: ^{1}H NMR spectrum of 4-(methacryloyloxy)piperidin-1-ium chloride

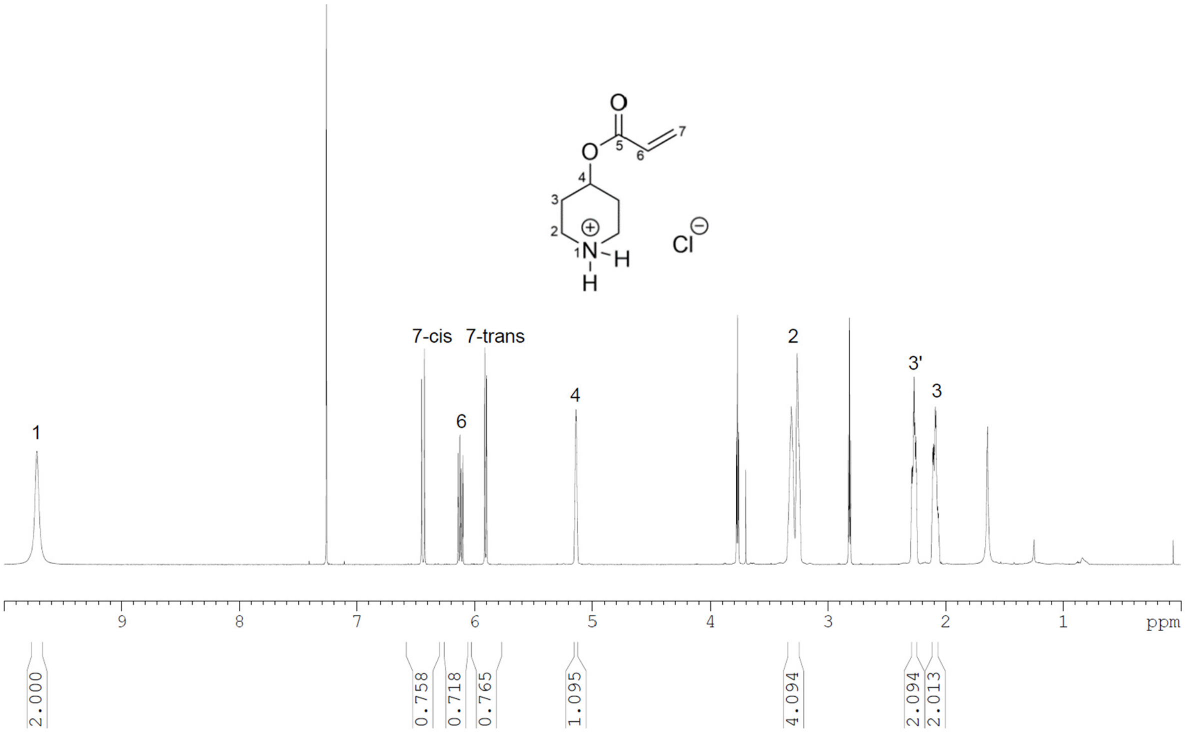

Figure S1: ^{1}H NMR spectrum of 4-(acryloyloxy)piperidin-1-ium chloride

Table S1: Different compositions of piperidin-4yl methacrylate and acrylate gels and polymerization parameters for bulk gels

Composition code	Catalytic monomer	Complementary monomer	Cross-linker	Solvent	Concentration (15 mmol in x mL)	UV irradiation time (s)	UV intensity (W)
A	**4** (90 %)	MMA (5 %)	EGDMA (5 %)	H_2O	0.8	20	0.42
B	**4** (50 %)	MMA (45 %)	EGDMA (5 %)	H_2O	0.6	40	0.84
C*	**4** (30 %)	MMA (65 %)	EGDMA (5 %)	H_2O	0.6	140	1.28
D*	**4** (20 %)	MMA (75 %)	EGDMA (5 %)	H_2O	0.6	140	1.28
E	**7** (90 %)	DMAA (5 %)	MBAM (5 %)	H_2O	0.8	20	0.42
F	**7** (50 %)	DMAA (45 %)	MBAM (5 %)	H_2O	0.6	15	0.42
G	**7** (20 %)	DMAA (75 %)	MBAM (5 %)	H_2O	0.6	10	0.42

* No gel formation

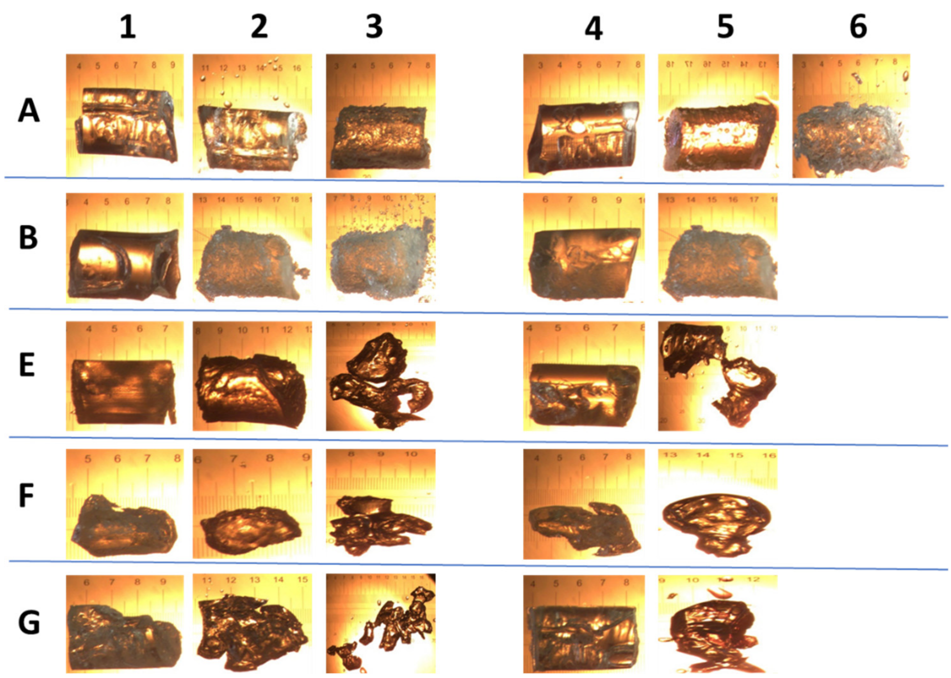

Figure S3: Optical microscopic images of cylindrical gels, **A**, **B**, **E**, **F** and **G** before and after swelling. Here, **1**, **2** and **3** indicated the swelling of the gels at room temperature for 0 h, 2 h and 24 h, respectively. The swelling of the gels at 40 ^{0}C indicated **4**, **5** and **6** for 0 h, 2 h and 24 h, respectively.

Table S2: Solubility tests of curcumin at room temperature and at 40 °C

mixture of solvents	water/ methanol		water/ ethanol		water/ isoprop.		DMSO/ water		DMSO/ methanol		DMSO/ ethanol		DMSO/ isoprop.	
ratio	7:3	3:7	7:3	3:7	7:3	3:7	7:3	3:7	7:3	3:7	7:3	3:7	7:3	3:7
solubility at RT	no	no	no	no	no	no	no	no	yes	yes	yes	yes	yes	yes
solubility at 40 °C	no	no	no	no	no	no	no	yes	-	-	-	-	-	-

Table S3: Swelling behaviour of gels in different solvents

solvent	observation
water	very high swelling of the gels; gels got disintegrated
methanol	swelling of gels; no disintegration
ethanol	no swelling; precipitations inside of the gel, opaque gels

Table S4: Percentages of solvent uptake after 2 h and 24 h of gels of composition A and B

Composition	Temperature	W_M [%] 2 h	W_M [%] 24 h
A	RT	3 ± 1	8 ± 2
A	40 °C	6 ± 3	-9 ± 5
B	RT	22 ± 5	25 ± 12
B	40 °C	11 ± 1	disintegrated
E	RT	33 ± 6	155 ± 48
E	40 °C	120 ± 5	disintegrated
F	RT	47 ± 48	40 ± 49
F	40 °C	120 ± 25	disintegrated
G	RT	7 ± 10	94 ± 17
G	40 °C	66 ± 27	disintegrated

Table S5: Conversion of batch reaction of 4-nitrobenzaldehyde with CUM and different contents of homogeneous catalyst PD

Entry No.	Reactants		Catalyst	T [°C]	t [h]	Conversion [%]
1	CUM 1 eq.	4-nitrobenzaldeyde 1 eq.	90 % PD	40	24	64
					48	55
					72	50
2	CUM 1 eq.	4-nitrobenzaldeyde 2 eq.	90 % PD	40	24	78
					48	95
					72	88
3	CUM 1 eq.	4-nitrobenzaldeyde 2 eq.	50 % PD	40	24	40
					48	79
					72	78
4	CUM 1 eq.	4-nitrobenzaldeyde 2 eq.	20 % PD	40	24	16
					48	32
					72	34
5	CUM 1 eq.	4-nitrobenzaldeyde 2 eq.	Without catalyst	40	24	-
					48	6
					72	7

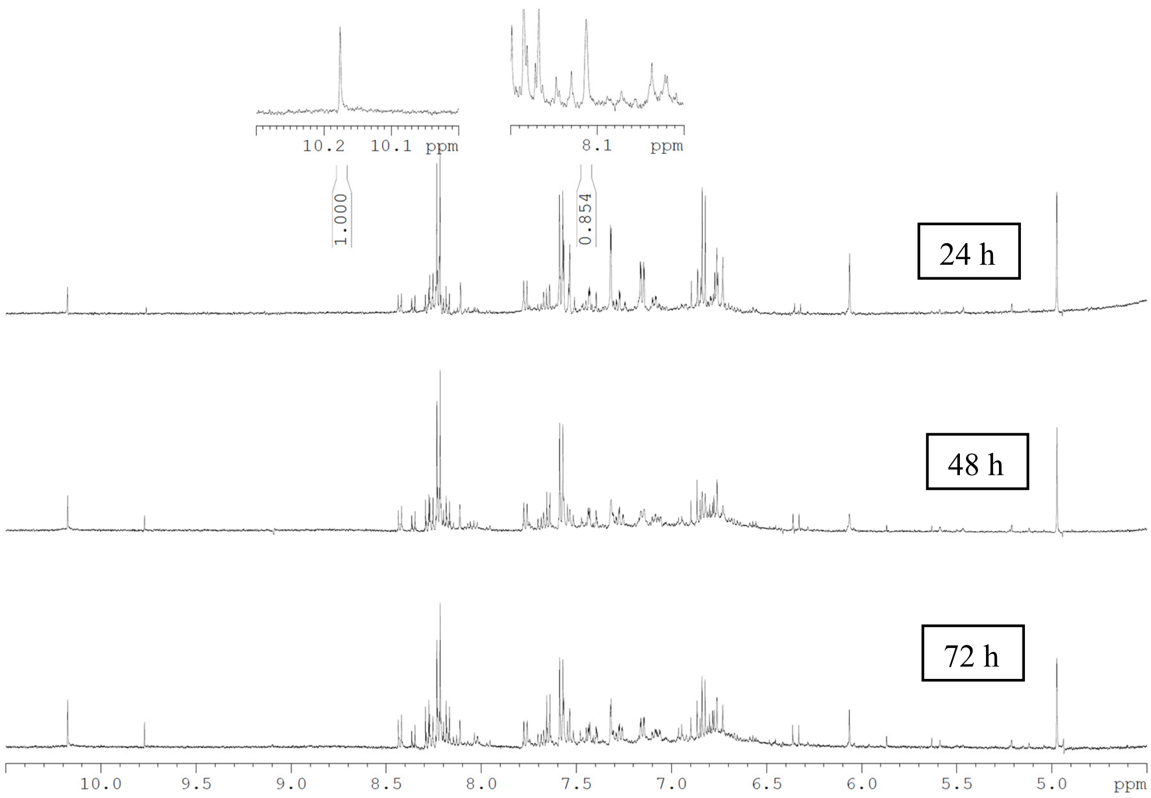

Figure S4: ^{1}H NMR spectra of entry number 1 in Table S4

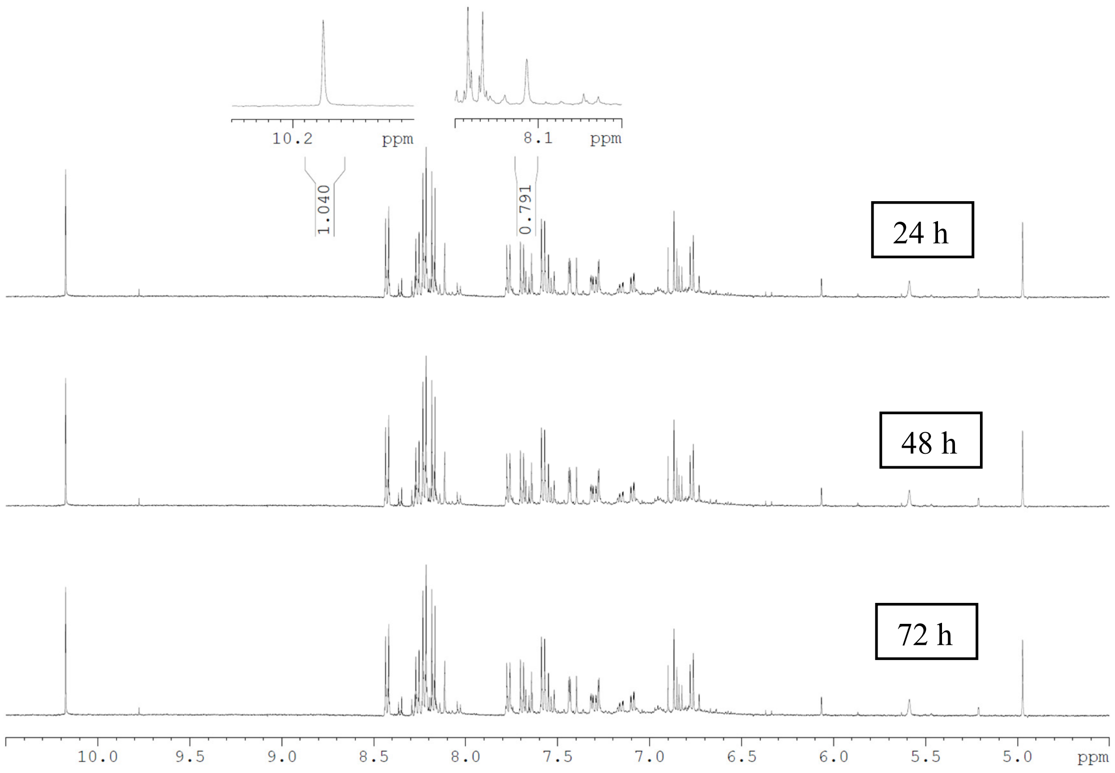

Figure S5: ^{1}H NMR spectra of entry number 2 in Table S4

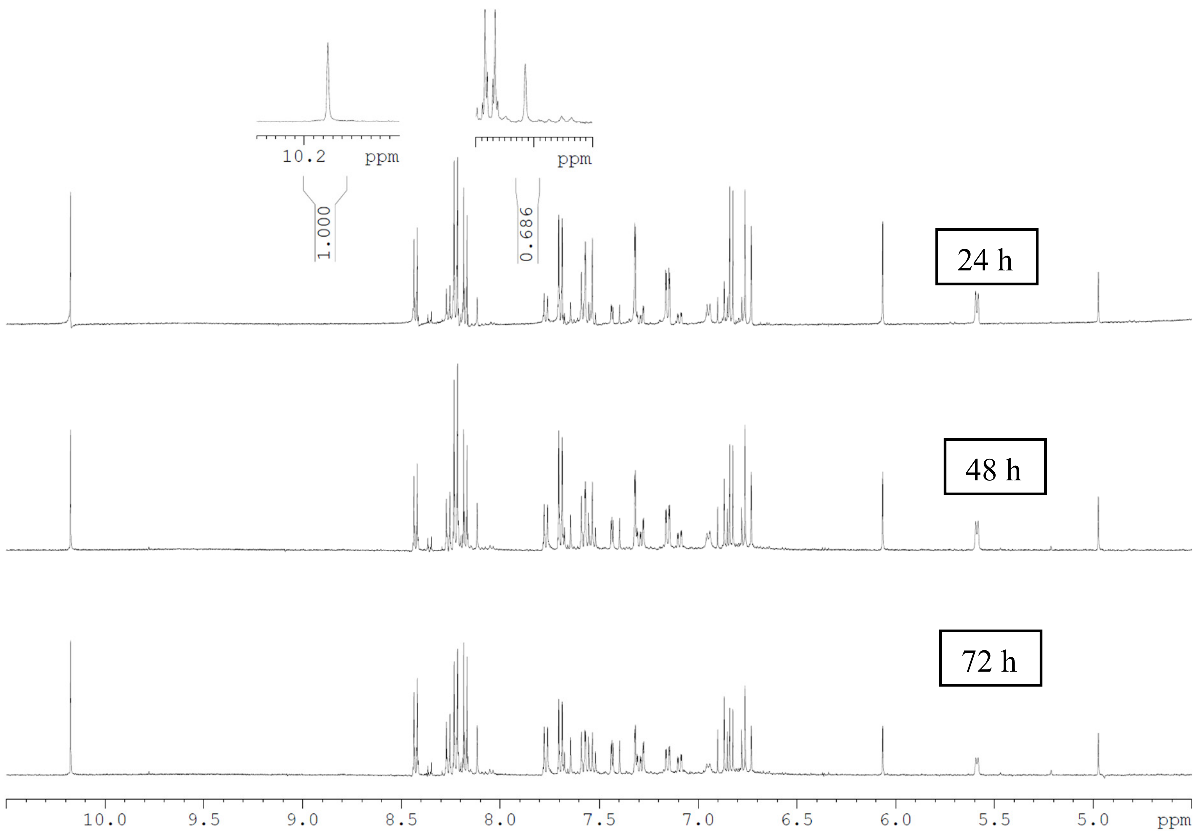

Figure S6: ^{1}H NMR spectra of entry number 3 in Table S4

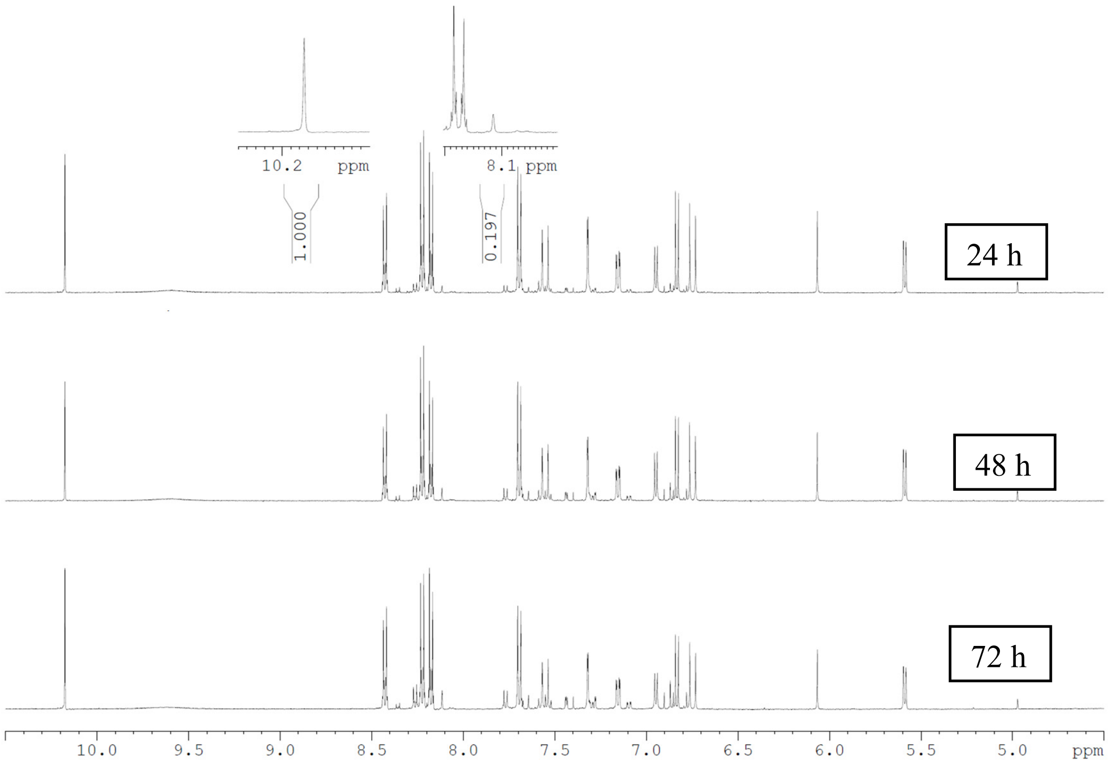

Figure S7: ^{1}H NMR spectra of entry number 4 in Table S4

Table S6: Conversion of heterogeneously catalysed batch reactions of CUM with 4-nitrobenzaldehyde catalysed by cylindrical gels of compositions A, B, E, F, and G

Entry No.	Reactants (1 : 2 eq.)		Catalyst, cylindrical gels (20 mg)	T [°C]	t [h]	Conversion [%]
1	CUM	4-nitrobenzaldehyde	E	20	24<br>48<br>72	-<br>-<br>-
2	CUM	4-nitrobenzaldehyde	E	40	24<br>48<br>72	33<br>54<br>82
3	CUM	4-nitrobenzaldehyde	F	40	24<br>48<br>72	25<br>47<br>61
4	CUM	4-nitrobenzaldehyde	G	40	24<br>48<br>72	11<br>21<br>32
5	CUM	4-nitrobenzaldehyde	A	40	24<br>48<br>72	9<br>12<br>34
6	CUM	4-nitrobenzaldehyde	B	40	24<br>48<br>72	91<br>>99<br>>99

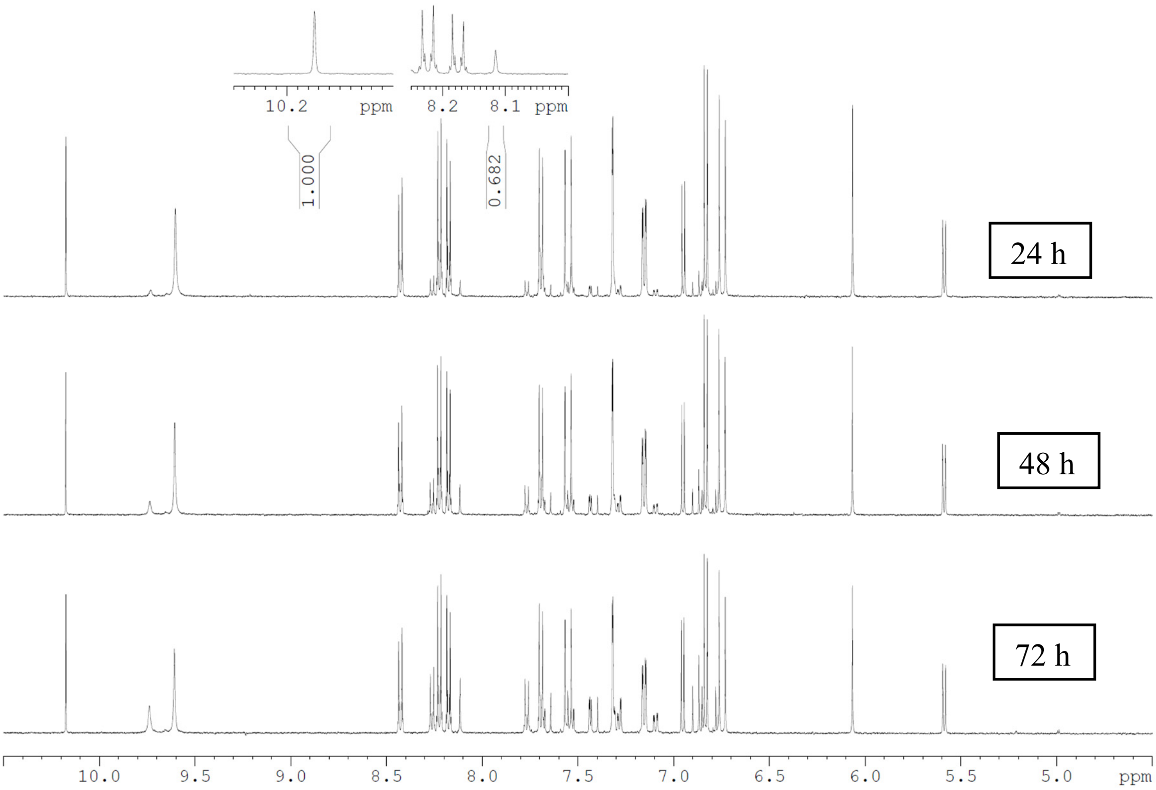

Figure S8: ^{1}H NMR spectra of entry number 2 in Table S5

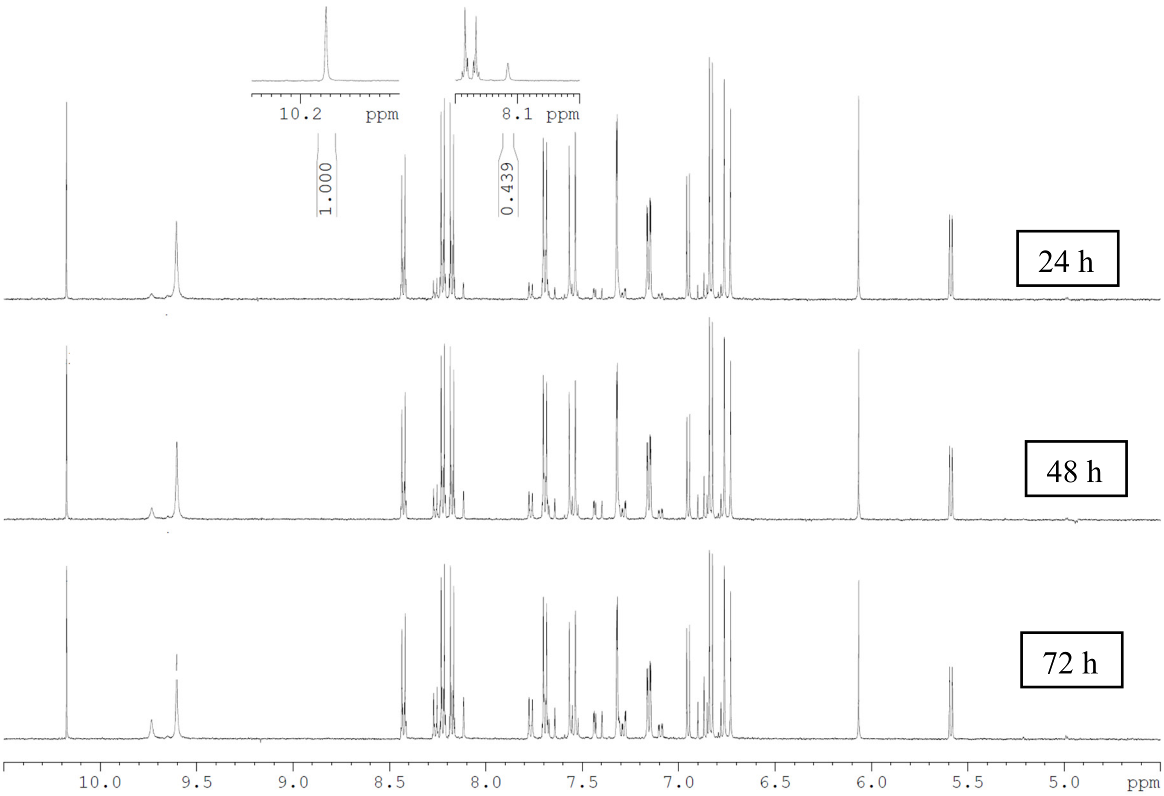

Figure S9: ^{1}H NMR spectra of entry number 3 in Table S5

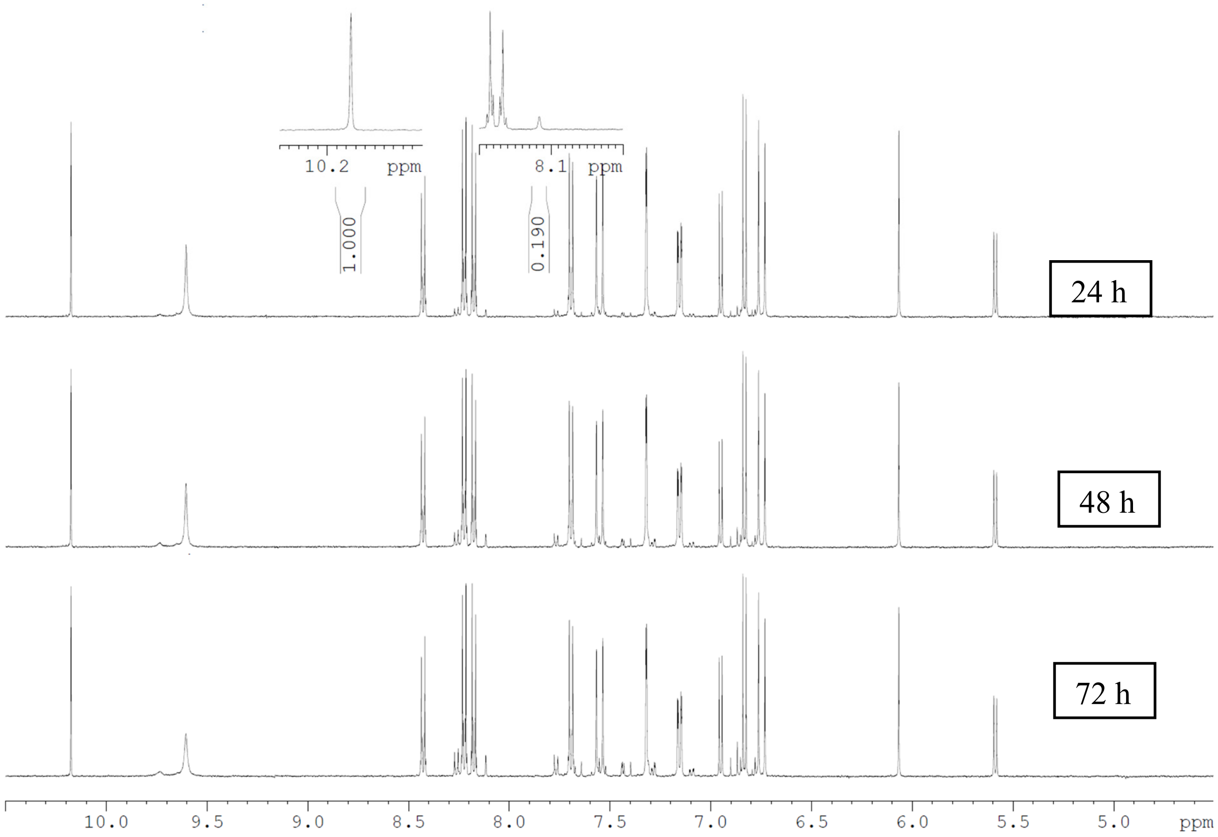

Figure S10: ^{1}H NMR spectra of entry number 4 in Table S5

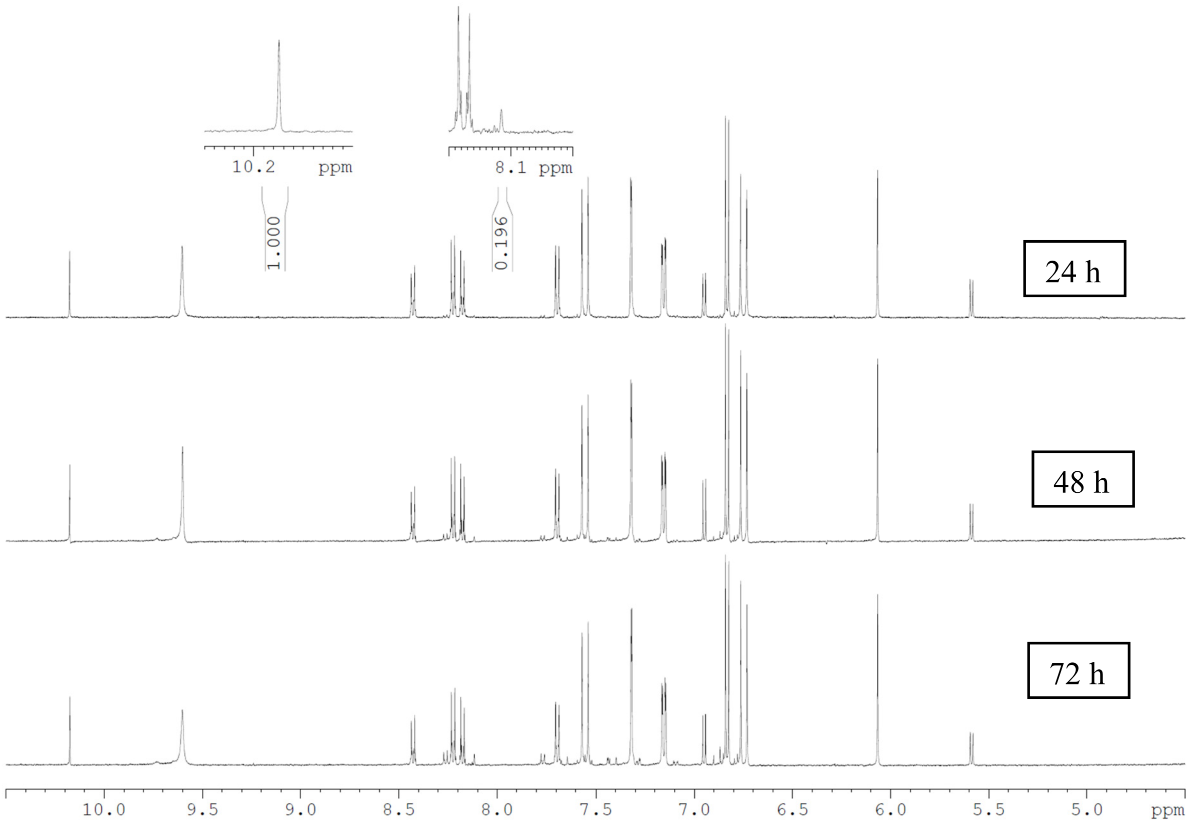

Figure S11: ^{1}H NMR spectra of entry number 5 in Table S5

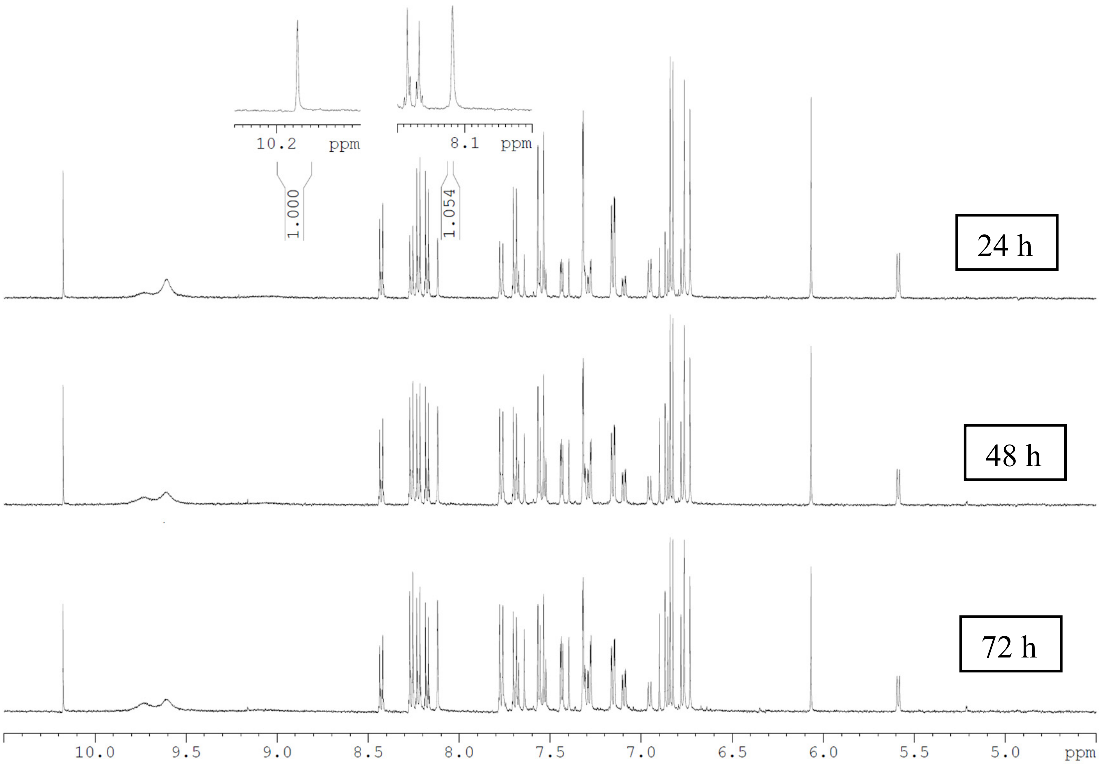

Figure S12: ^{1}H NMR spectra of entry number 6 in Table S5

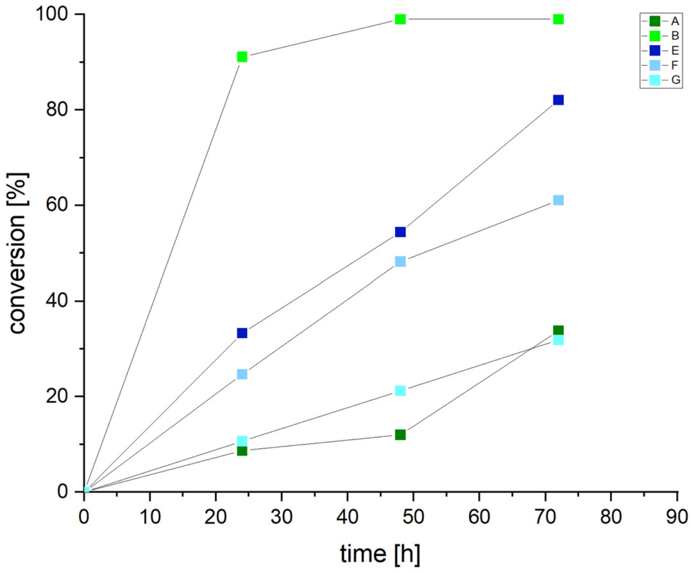

Figure S13: Conversions at different times of batch reactions of curcumin (CUM) with 4-nitrobenzaldehyde catalysed with gels of the compositions A, B, E, F, and G.

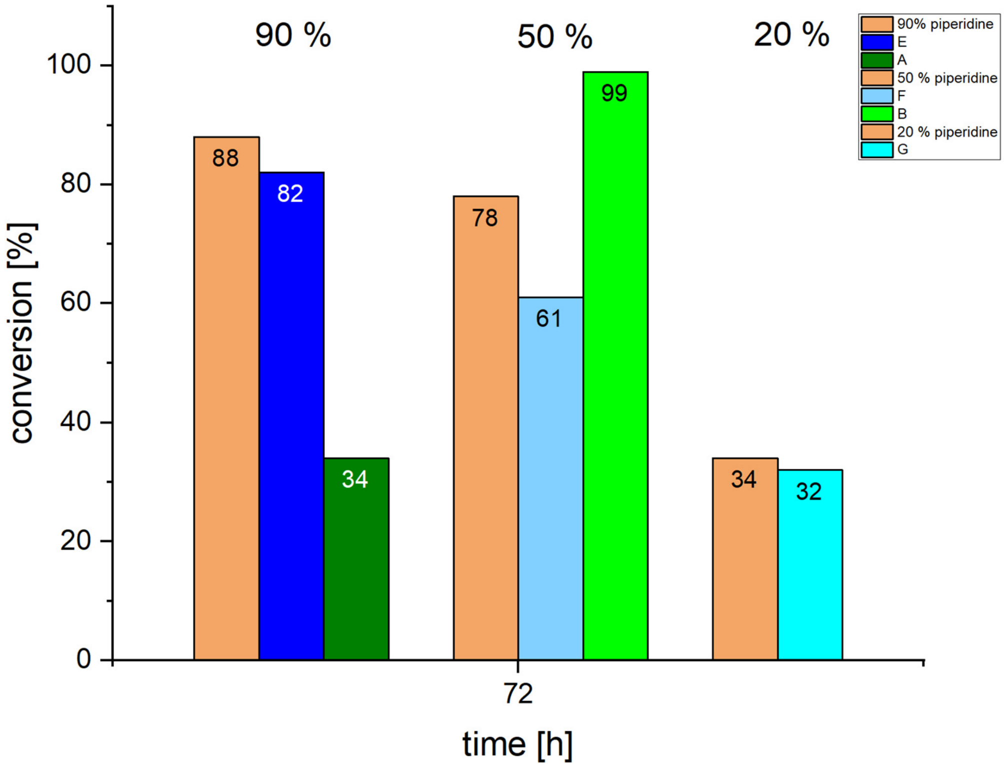

Figure S14: Graphical comparison of conversion in batch reactions, which were homogenously catalysed with piperidine (PD) and heterogeneously catalysed with cylindrical gels

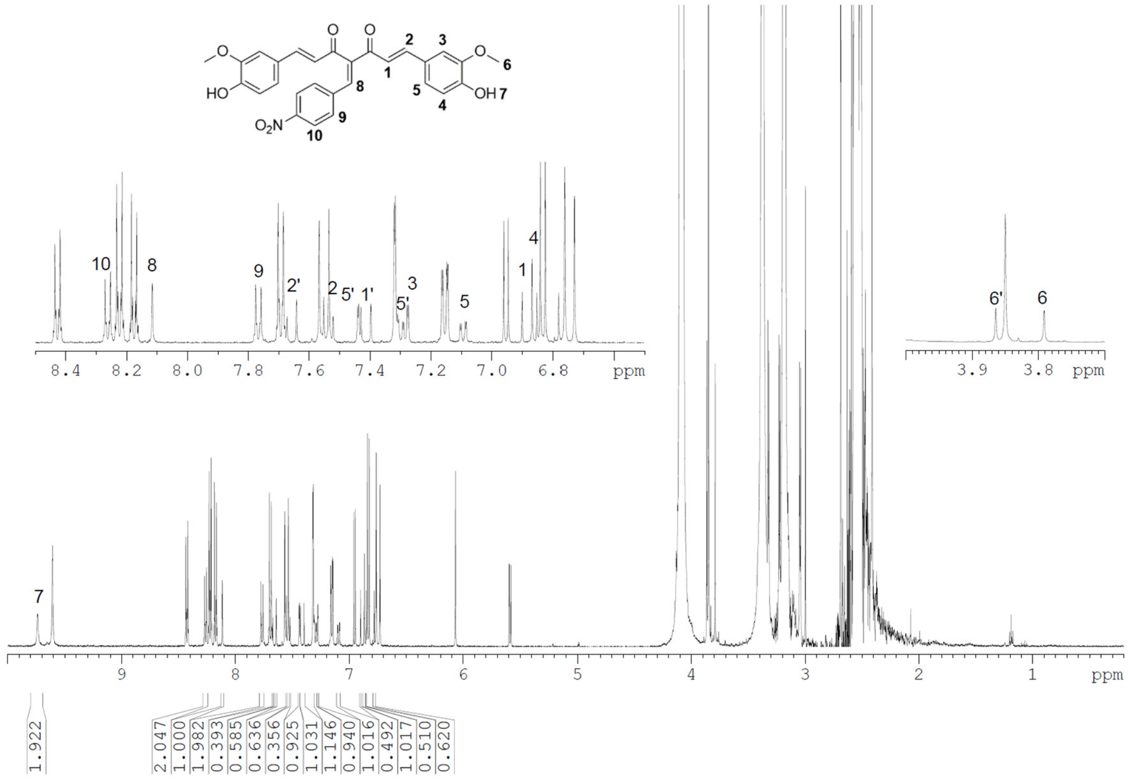

Figure S15: ^{1}H NMR spectrum of (1E,6E)-1,7-bis(4-hydroxy-3-methoxyphenyl)-4-(4-nitrobenzylidene)hepta-1,6-diene-3,5-dione

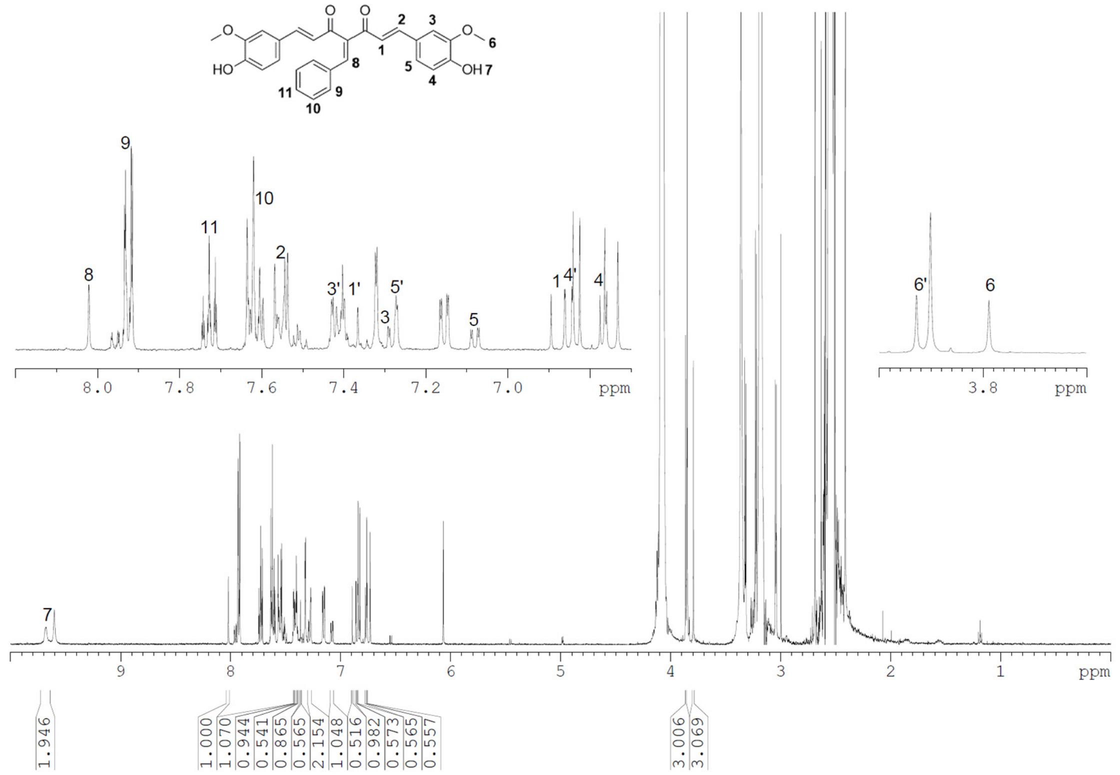

Figure S16: 1H NMR spectrum of (1E,6E)-4-benzylidene-1,7-bis(4-hydroxy-3-methoxyphenyl)hepta-1,6-diene-3,5-dione

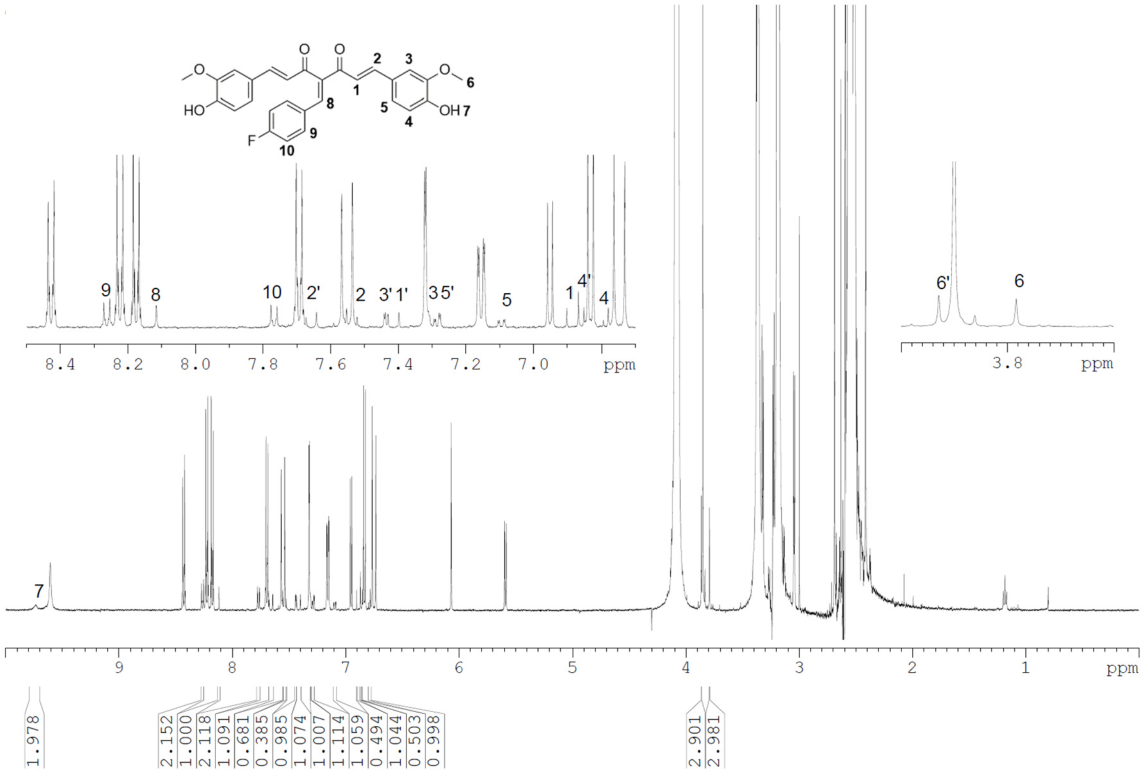

Figure S17: ^{1}H NMR spectrum of (1E,6E)-4-(4-fluorobenzylidene)-1,7-bis(4-hydroxy-3-methoxyphenyl)hepta-1,6-diene-3,5-dione

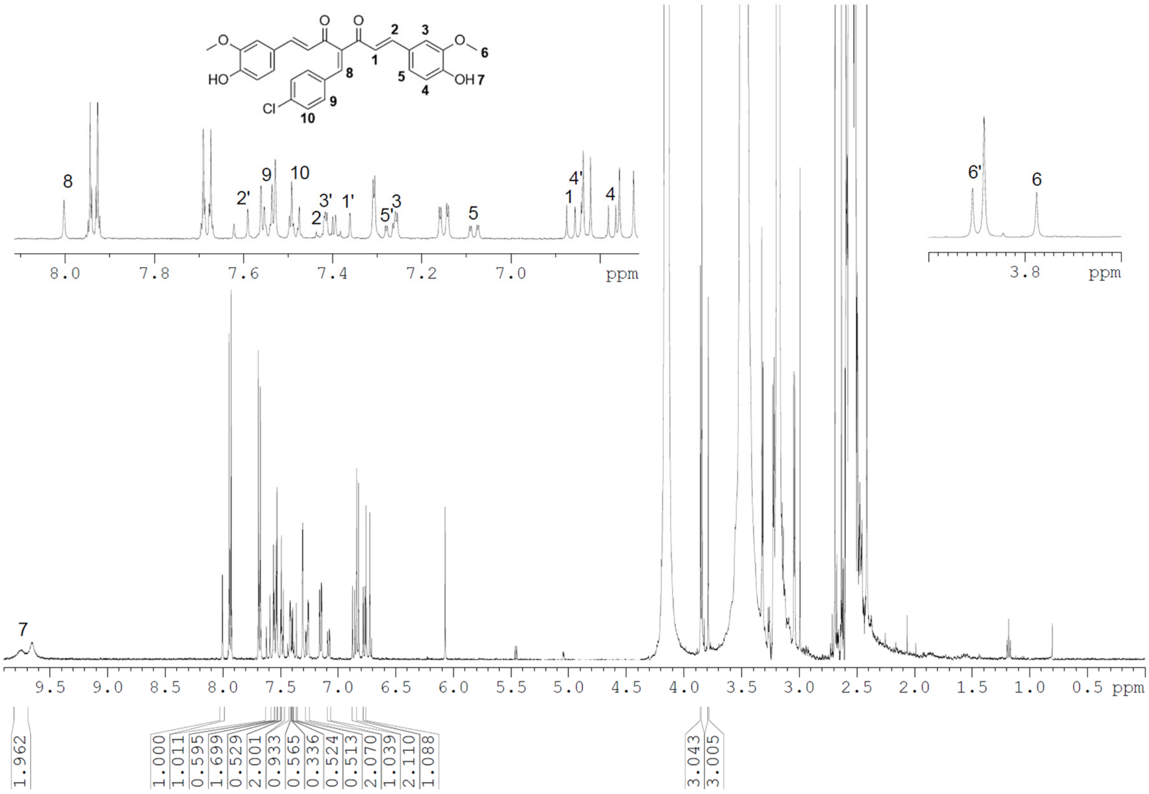

Figure S18: ^{1}H NMR spectrum of (1E,6E)-4-(4-chlorobenzylidene)-1,7-bis(4-hydroxy-3-methoxyphenyl)hepta-1,6-diene-3,5-dione

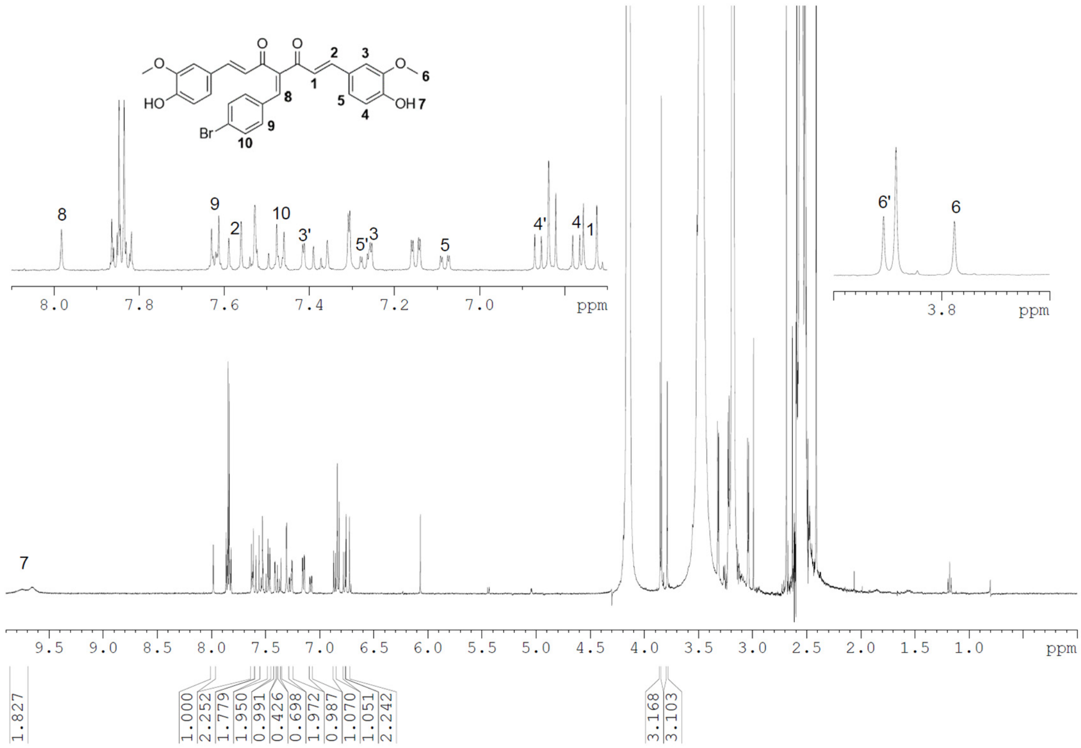

Figure S19: ^{1}H NMR spectrum of (1E,6E)-4-(4-bromobenzylidene)-1,7-bis(4-hydroxy-3-methoxyphenyl)hepta-1,6-diene-3,5-dione

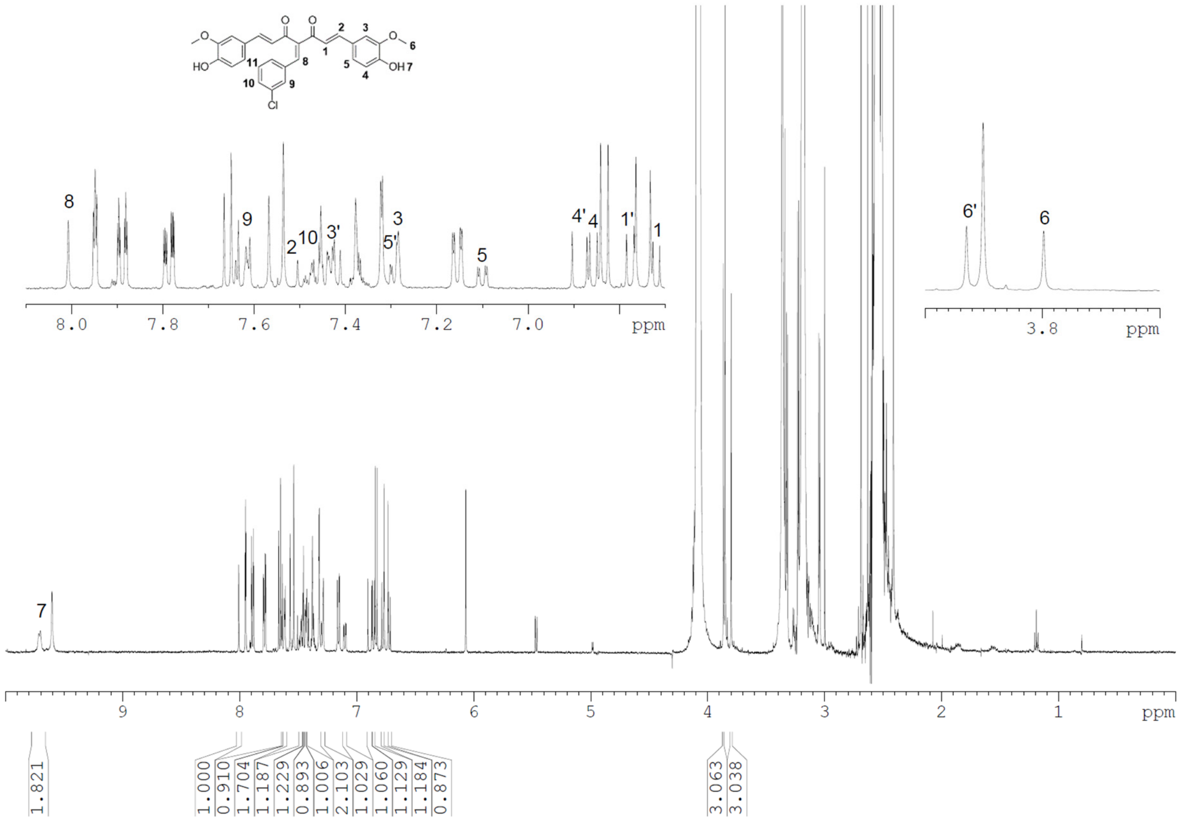

Figure S20: ^{1}H NMR spectrum of (1E,6E)-4-(3-chlorobenzylidene)-1,7-bis(4-hydroxy-3-methoxyphenyl)hepta-1,6-diene-3,5-dione

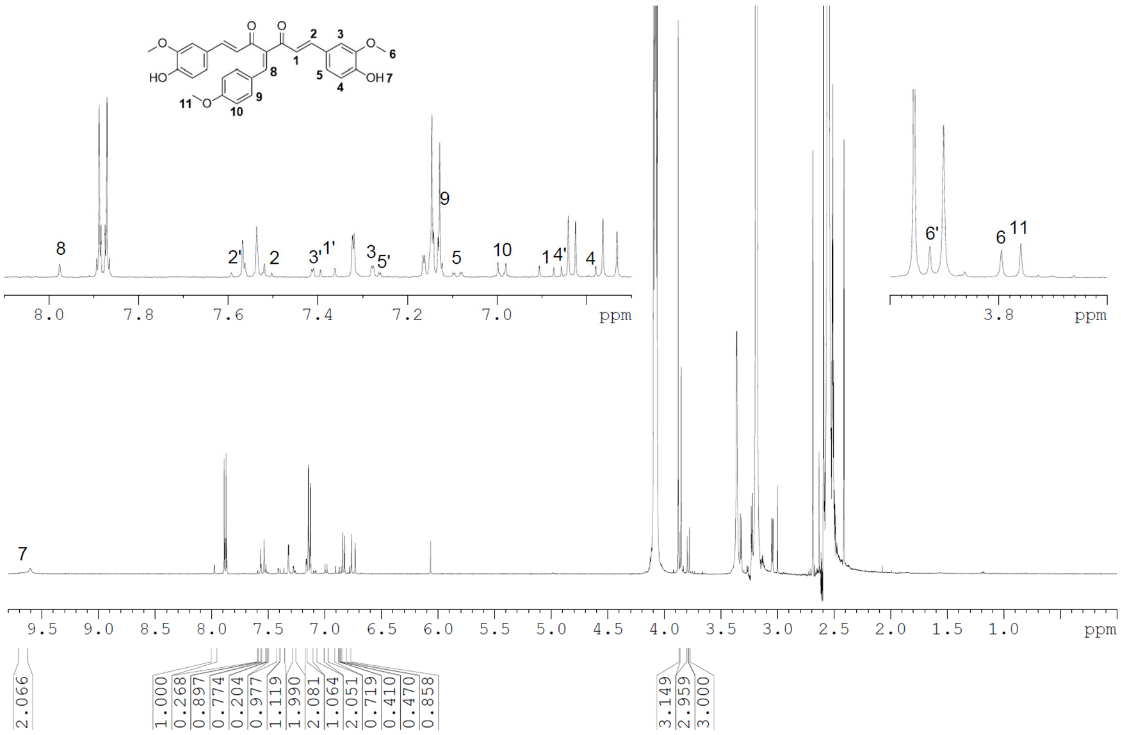

Figure S21: ^{1}H NMR spectrum of (1E,6E)-1,7-bis(4-hydroxy-3-methoxyphenyl)-4-(4-methoxybenzylidene)hepta-1,6-diene-3,5-dione

Table S7: Conversion of microflow reactions of CUM with 4-nitrobenzaldehyde catalysed by 662 gel dots of compositions A, B, E, F, and G

Entry No.	Reactants (1 :2 eq.)		Catalyst, gel dots (662 number) composition	T [°C]	t [h]	Conversion [%]
1	CUM	4-nitrobenzaldehyde	E	40	24<br>48<br>72	-<br>28<br>22
2	CUM	4-nitrobenzaldehyde	F	40	24<br>48<br>72	-<br>70<br>72
3	CUM	4-nitrobenzaldehyde	G	40	24<br>48<br>72	24<br>43<br>32
4	CUM	4-nitrobenzaldehyde	A	40	24<br>48<br>72	-<br>33<br>48
5	CUM	4-nitrobenzaldehyde	B	40	24<br>48<br>72	-<br>-<br>-

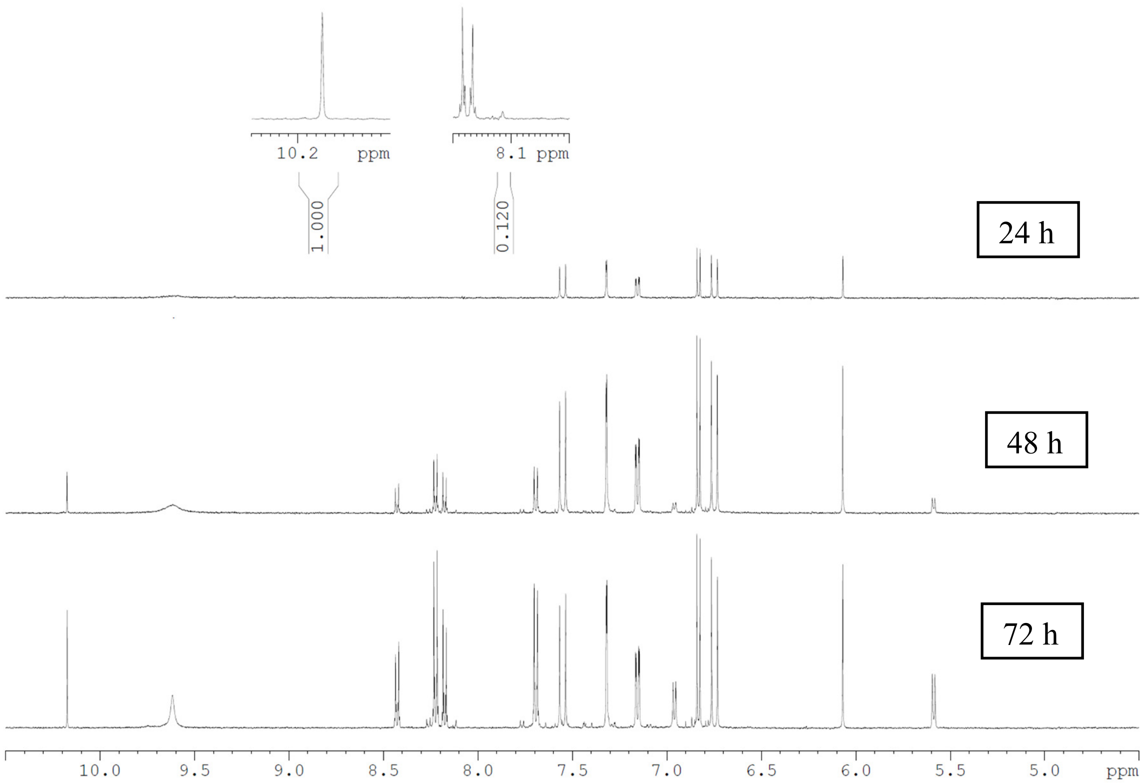

Figure S22: ^{1}H NMR spectra of entry number 1 in Table S6

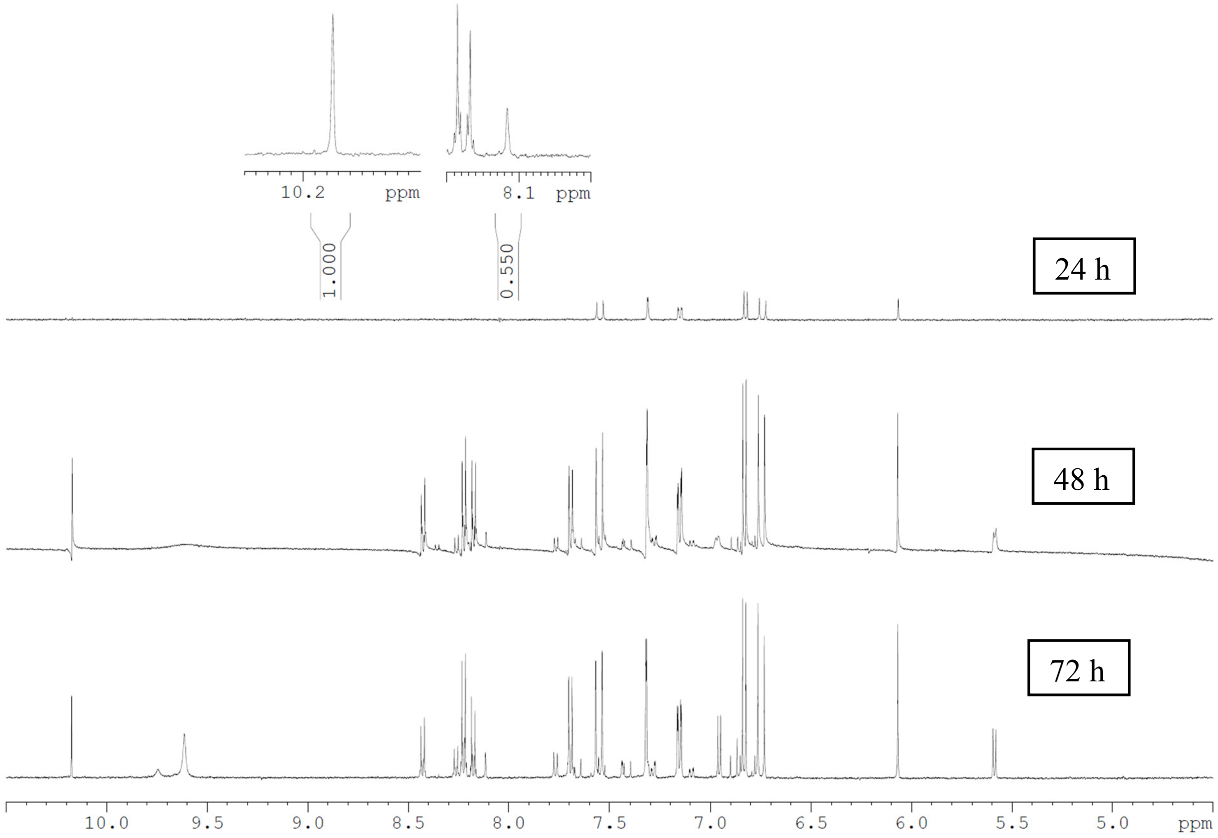

Figure S23: ^{1}H NMR spectra of entry number 2 in Table S6

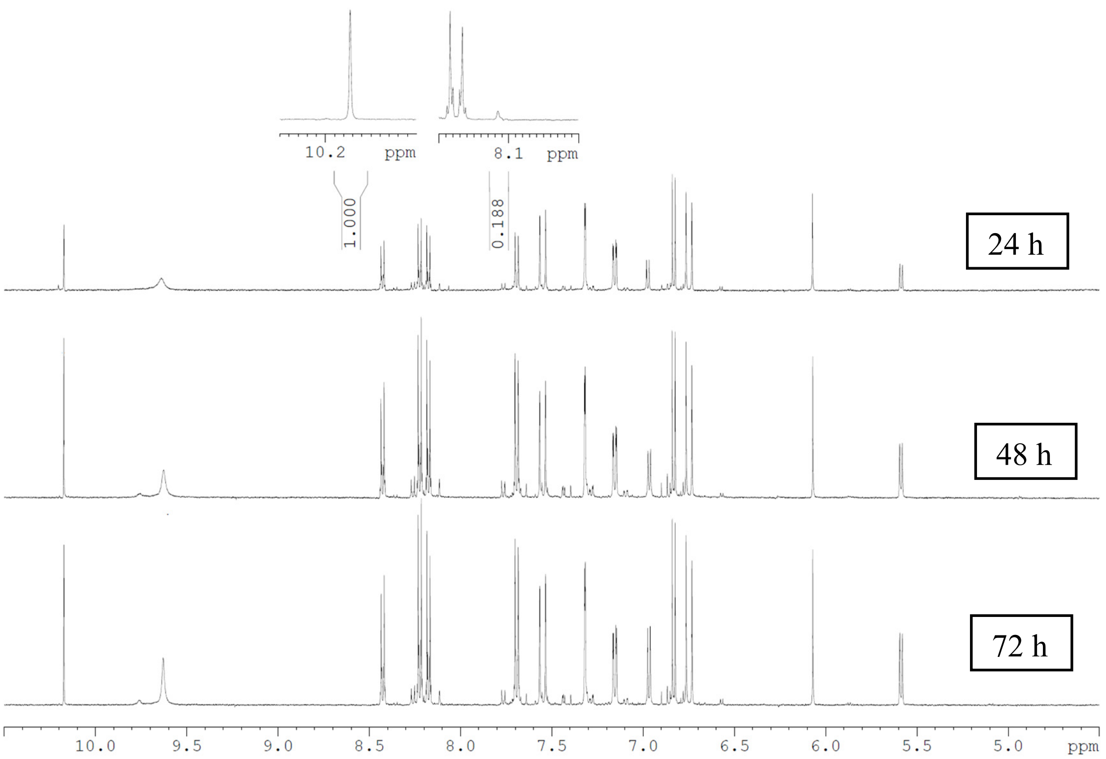

Figure S24: ^{1}H NMR spectra of entry number 3 in Table S6

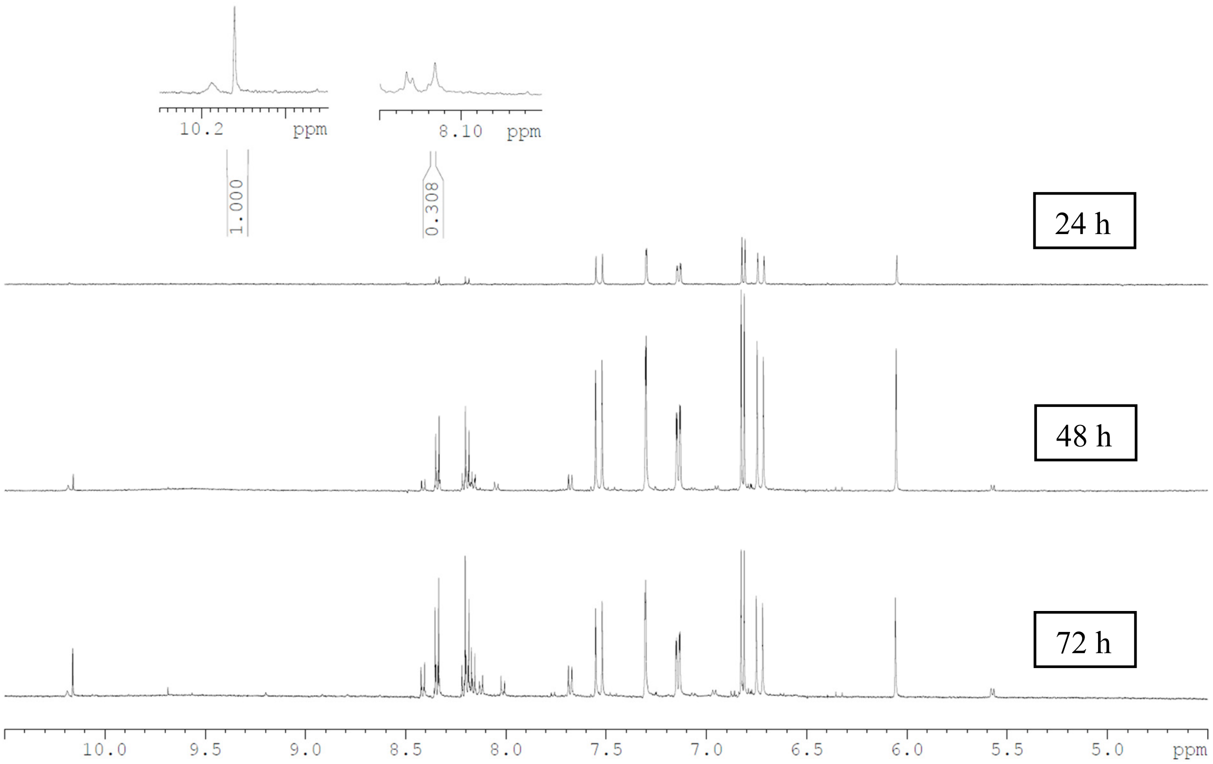

Figure S25: ^{1}H NMR spectra of entry number 4 in Table S6

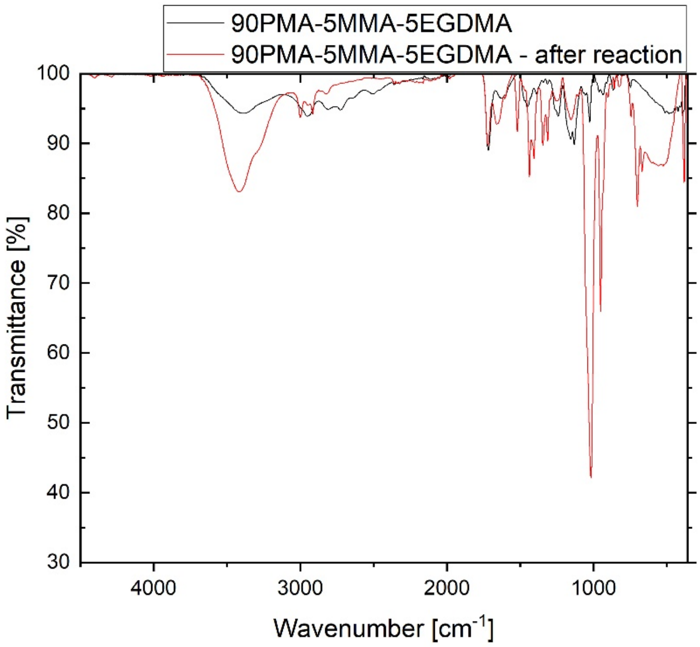

Figure S26: FTIR spectra of gels of composition A before and after catalysis of reactions of CUM with 4-nitrobenzaldehyde

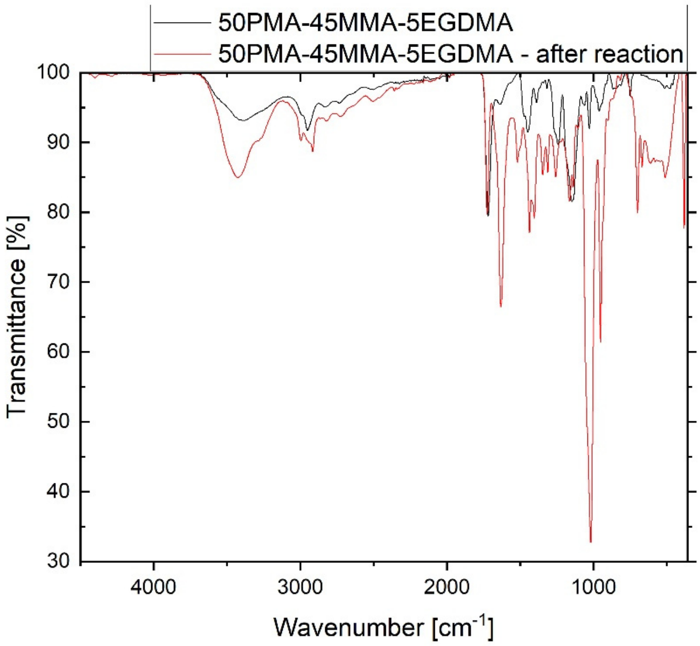

Figure S27: FTIR spectra of gels of composition B before and after catalysis of reactions of CUM with 4-nitrobenzaldehyde

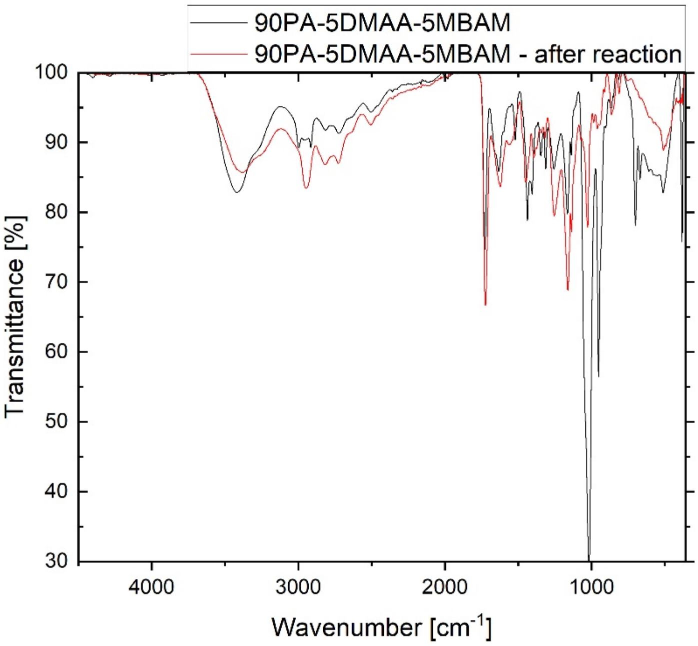

Figure S28: FTIR spectra of gels of composition E before and after catalysis of reactions of CUM with 4-nitrobenzaldehyde

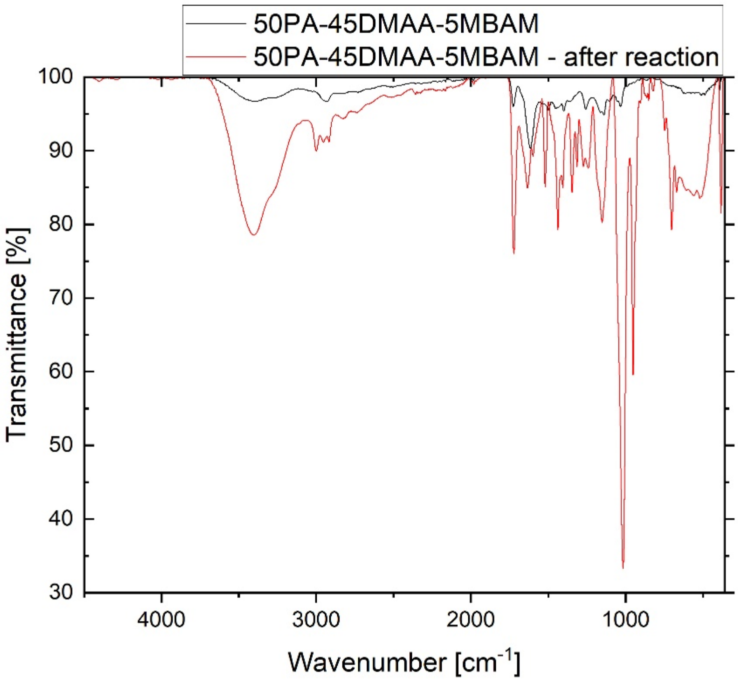

Figure S29: FTIR spectra of gels of composition F before and after catalysis of reactions of CUM with 4-nitrobenzaldehyde

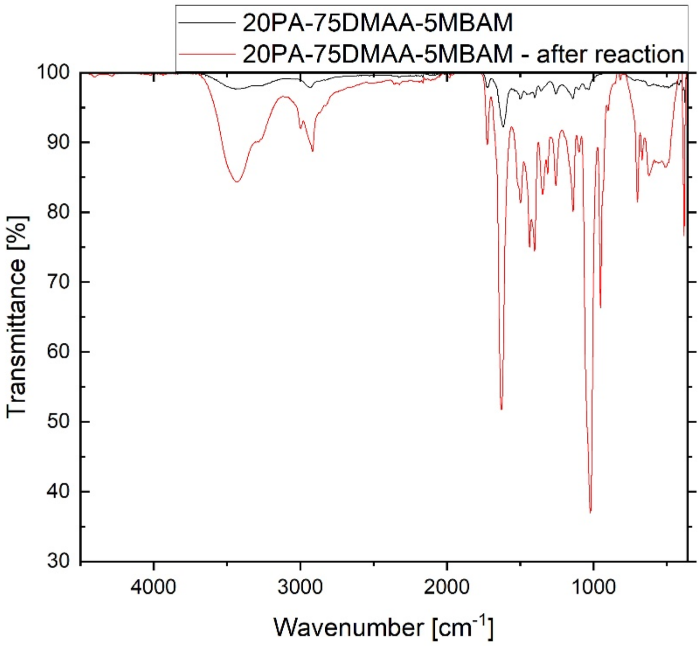

Figure S30: FTIR spectra of gels of composition G before and after catalysis of reactions of CUM with 4-nitrobenzaldehyde

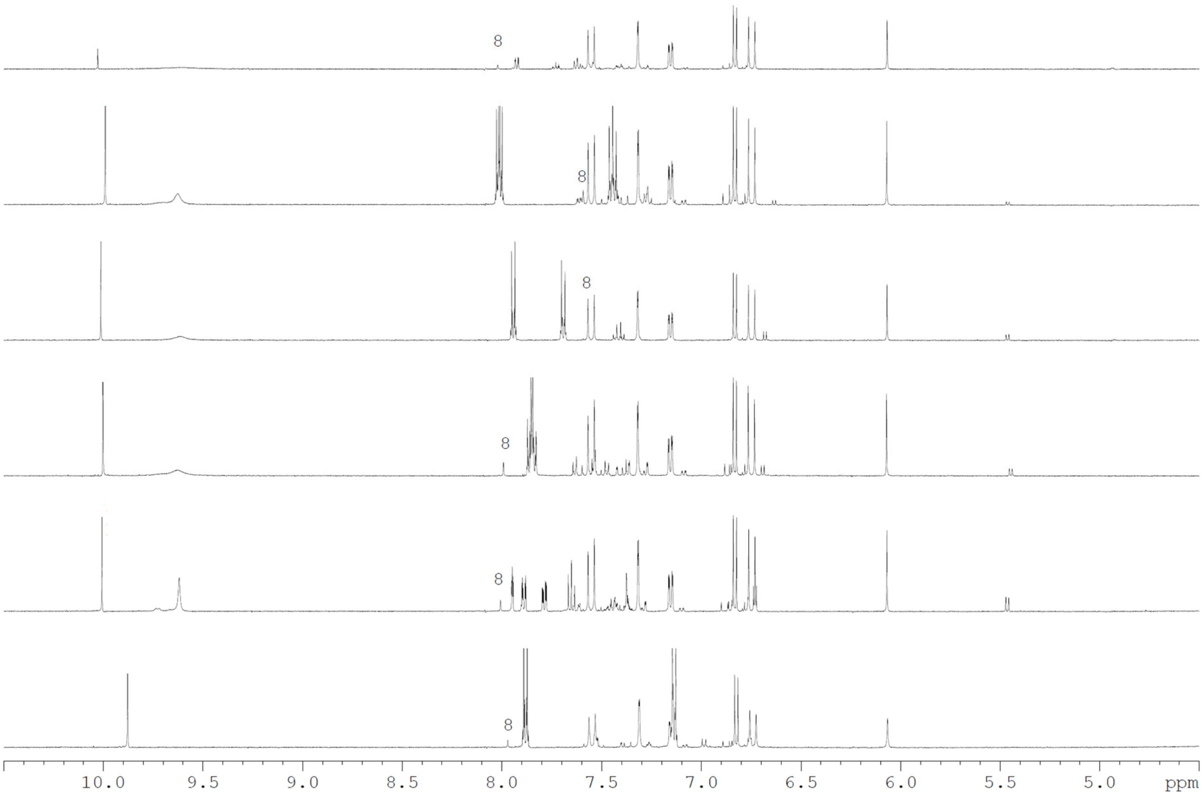

Figure S31: ^{1}H NMR spectra of curcumin derivatives synthesized in the MFR using gel dots composition F for 72 h.
